# VEGA is an interpretable generative model for inferring biological network activity in single-cell transcriptomics

**DOI:** 10.1038/s41467-021-26017-0

**Published:** 2021-09-28

**Authors:** Lucas Seninge, Ioannis Anastopoulos, Hongxu Ding, Joshua Stuart

**Affiliations:** Department of Biomolecular Engineering and Genomics Institute, University of California, Santa Cruz, CA USA

**Keywords:** Computational models, Machine learning, Software, Regulatory networks

## Abstract

Deep learning architectures such as variational autoencoders have revolutionized the analysis of transcriptomics data. However, the latent space of these variational autoencoders offers little to no interpretability. To provide further biological insights, we introduce a novel sparse Variational Autoencoder architecture, VEGA (VAE Enhanced by Gene Annotations), whose decoder wiring mirrors user-provided gene modules, providing direct interpretability to the latent variables. We demonstrate the performance of VEGA in diverse biological contexts using pathways, gene regulatory networks and cell type identities as the gene modules that define its latent space. VEGA successfully recapitulates the mechanism of cellular-specific response to treatments, the status of master regulators as well as jointly revealing the cell type and cellular state identity in developing cells. We envision the approach could serve as an explanatory biological model for development and drug treatment experiments.

## Abstract (web summary)

Developing interpretable models is a major challenge in single cell deep learning. Here we show that the VEGA variational autoencoder model, whose decoder wiring mirrors gene modules, can provide direct interpretability to the latent space further enabling the inference of biological module activity.

## Introduction

Recent advances in single-cell RNA sequencing (scRNA-Seq) technologies have enabled the characterization of cellular states at an unprecedented scale and resolution^1^. Among the many widely-used frameworks for analyzing complex transcriptomic patterns in single cells, artificial neural networks (ANNs) such as autoencoders (AEs)^2^ have emerged as powerful tools. AEs are neural networks that transform an input dataset into a decoded representation while minimizing the information loss^3^. The diversity in their architectural design makes AEs suitable to tackle various important challenges of scRNA-Seq analysis, such as dimensionality reduction^4^, clustering^5^, and data denoising^6^.

More recently, deep generative models such as variational autoencoders^7^ (VAEs) have proven to be extremely useful for the probabilistic modeling of single-cell transcriptomes, such as scVI and scGen^8–10^. While standard AEs learn to reconstruct an input dataset, deep generative architectures explicitly model and learn the true data distribution, which allows a broader set of queries to be addressed. While deep generative models have shown impressive performance for their dedicated modeling tasks, they often lack interpretability thus cannot offer a biologically meaningful latent representation of transcriptomes. For example, latent perturbation vectors extracted with scGen cannot be directly related to gene module variations^10^.

Integration of prior knowledge about gene modules to aid interpretability has already been successfully applied to transcriptomics data. DCell^11^ is a deep neural network integrating the hierarchical information about the molecular subsystems involved in cellular processes to guide supervised learning tasks, such as predicting growth in yeast. Such a model yields an informative biological interpretation of predictions by investigating the activation of the different subsystems embedded in the model’s architecture. However, this model only works in a supervised learning setting where the goal is to predict a phenotypic outcome. On the other hand, f-scLVM^12^ is a Bayesian hierarchical model with explicit prior biological knowledge specification to infer the activity of latent factors as a priori characterized gene modules. While this approach enables the modeling of single-cell transcriptomes in an interpretable manner, the computational cost of the inference algorithm, as well as the absence of inference for out-of-sample data, make the development of more efficient approaches highly desirable.

Here we propose VEGA (VAE enhanced by gene annotations), a VAE with a sparse linear decoder informed by biological networks. VEGA offers an interpretable latent space to represent various biological information, e.g., the status of biological pathways or the activity of transcriptional regulators. Specifically, the scope of VEGA is twofold, (1) encoding data over an interpretable latent space and (2) inferring gene module activities for out-of-sample data.

## Results

### Architectural design of VEGA

**Fig. 1:**
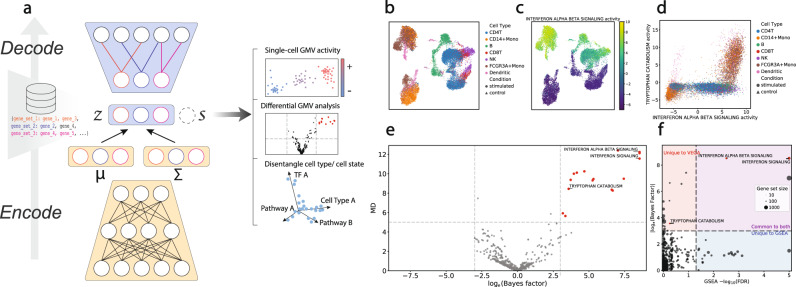
Designing a novel VAE architecture with interpretable latent space. **a** Overview of the VEGA model. Composed of a deep nonlinear encoder (*μ*, Σ) and a masked linear decoder, VEGA represents single-cell transcriptomics data into a lower-dimensional interpretable latent space *z* that approximates a set of user-supplied gene modules (GMV). Additionally, VEGA can integrate batch information as another variable *s* to condition its generative process on batch labels. **b** UMAP embedding of the latent space of VEGA retains the biological signal of the Kang et al. PBMCs dataset^17^. **c** Inferred interferon-alpha/beta signaling pathway activity segregates stimulated cells from the control population. **d** Bivariate GMV plot showing the ability of the model to recover the tryptophan catabolism activity, an innate (Dendritic cells, FCGR3A+monocytes, CD14+monocytes) immune cell-specific response to the perturbation. **e** Volcano plot showing differentially active GMVs between stimulated and control innate immune cells. The red dots indicate GMVs with ∣log_e_(Bayes Factor)∣ > 3 and a mean absolute difference (MD) in the latent space of at least 5. **f** Comparison of VEGA Bayes Factor with GSEA -log_10_(FDR). The size of the dots indicates the gene set size. The red, blue, and purple quadrants correspond respectively to significant hits unique to our model, unique to GSEA, and common to both.

To create a readily interpretable VAE, we propose a novel architecture we refer to as VEGA (VAE enhanced by gene annotations) where the decoder (generative part) connections of the neural network are guided by gene module membership as recorded in gene annotation databases (e.g., Gene Ontology, PANTHER, MolSigDB, or Reactome) (Fig. 1a). In many standard VAE implementations, the information bottleneck of the encoder-decoder architecture often represents latent variables modeled as a multivariate normal distribution. Despite providing highly informative representations of the input data, VAE latent variables are in general hard to interpret. Svensson et al.^13^ proposed using a linear decoder which directly connects latent variables to genes, providing interpretability similar to that offered by standard factor models such as PCA. Although providing valuable insights, such an approach requires further statistical enrichment tests on the weights of the decoder to infer biological processes contributing to the single-cell expression dataset.

In contrast to previous approaches, VEGA implements a sparse architecture that explicitly reflects knowledge about gene regulation. In the service of biological pathways, genes work together in gene modules, regulated by common transcription factors that often produce correlated expression. Thus, if a given scRNA-Seq dataset *X* reflects the patterns of known gene modules, then it is possible for a VAE to learn a compact representation of the data by incorporating those modules as latent variables *Z*. VAEs use multiple layers to approximate the latent variable distribution and produce a low dimensional, nonlinear representation of the original feature data. Importantly, the first and last layers directly connect to the input or predicted features and so can be fashioned to depict intuitive groupings. Standard VAEs use a fully connected layer for both the encoding first layer and the decoding final layer (SFig. 1aiv). Instead, VEGA uses a gene membership mask **M** to select a subset of trainable weights in the decoder layer that are determined by a given set of gene modules (see Methods). The mask is applied to the weights that connect to the predicted output features to yield an interpretation of the latent variable layer where each latent variable is viewed as a specific gene module, henceforth referred to as a gene module variable (GMV). Specifically, the generative part of VEGA (decoder) maintains a link from a GMV to an output gene only if this gene is annotated to be a member of this specific gene module. The two main advantages of this design are (1) the latent variables are directly interpretable as the activity of biological modules and (2) the flexibility in the gene module specification allows it to generalize to different biological abstractions (such as pathways, gene regulatory networks (GRNs), or even cell types) and can be taken from any of several curated databases of gene sets (such as MSigDB^14^, Reactome pathways^15^, inferred GRNs^16^). Additionally, VEGA incorporates information about covariates such as technical replicates in its latent space. This can be used to alleviate batch effects, as it has been demonstrated in previous deep generative models for single-cell data^9^ (Fig. 1a and SFig. 2)

Note that it is possible to implement gene module sparseness in the encoder half of the neural network (inference part), in addition to (or in place of) the decoder half (generative part), which gives three possible VAE architectures that we considered for single-cell RNA-seq analysis (SFig. 1ai–iii). As expected, we found that the GMV-guided designs resulted in decent although slightly worse performance compared to the full architecture (SFig. 1c). Among these options, we chose the sparse decoding architecture over the others for its improved separation of known cellular states and types in the Kang et al. PBMC data^17^ (SFig. 1b). Intuitively, using a deep encoder maintains a full VAE’s inference capacity to capture a potentially complex latent space while together with a sparse decoder approximates the posterior distribution of GMV activities *p*(*Z*∣*X*) to provide interpretation over gene modules. Additionally, we found that VEGA benefits from having a trainable, sparse decoder to adequately capture the biological signal of a dataset compared to simpler pathway transformations (SFig. 3).

### Recapitulating biological information over an interpretable latent space

We asked if VEGA could recapitulate the status of biological pathways by applying it to a published and well-studied peripheral blood mononuclear cells (PBMCs) dataset stimulated with the chemokine interferon-*β*^17^ (Methods). We first found that VEGA is able to capture cell types and stimulation status using the Reactome collection of processes and pathways^15^ in the GMV decoding layer (Fig. 1b). Specifically, we found that the interferon-*α*/*β* signaling GMV activity segregates stimulated and naive cells, confirming the ability of VEGA to capture pathway activity in its latent space (Fig. 1c, d). We further examined other known biological pathways involved in interferon-induced immune cell activation and found cell-type-specific activation of certain cellular processes. For example, tryptophan catabolism response to interferon separates innate immune cells (Dendritic cells, FCGR3A+monocytes, and CD14+monocytes) from adaptive immune cells (NK cells, T-cell CD8, T-cell CD4, and B cells) (Fig. 1d), as previously investigated^18,19^. Together, these results suggest that VEGA’s GMV’s reflect the expected major biological pathways in PBMCs and therefore may be useful for other datasets to project cells into an interpretable space, allowing investigation of cell-type-specific patterns at the cellular process level.

We next asked whether the differential activities of the GMVs accurately contrast pathway states as a function of a specific, experimentally controlled context.

For this purpose, we propose a similar Bayesian hypothesis testing procedure as introduced by Lopez et al.^9^ to study the difference in GMV activities. As VEGA models the posterior distribution of each GMV, we can formulate mutually exclusive hypotheses similar to differential gene expression tests (i.e., GMVs are activated at different levels). We can approximate the posterior probability of these hypotheses through Monte Carlo sampling of VEGA’s latent variable distribution. The ratio of hypothesis probabilities corresponds to the Bayes Factor^20^ (BF, see Methods).

When applied to innate immune cells in the stimulated vs control groups of the Kang et al.^17^ dataset, the BF analysis found GMVs that correspond to pathways expected to be activated in the stimulated groups (interferon signaling, tryptophan catabolism; ∣log_e_(BF)∣ > 3, Fig. 1e). We compared the GMV BFs with the false discovery rate (FDR) values of the standard GSEA toolkit (Methods, Fig. 1f). While both methods found the expected activation of the interferon-*α*/*β* signaling pathway GMV in the stimulated groups, GSEA missed the tryptophan catabolism activation in innate immune cells (Fig. 1f). Overall, VEGA seems more robust than GSEA to gene set size bias (Fig. 1f and SFig. 4), suggesting it may emphasize more context-relevant pathways. Additionally, the differential GMV activity test can be applied in a cell-type-specific fashion (similar to one-vs-rest differential gene expression analyses). We found that such a procedure yields informative results in terms of cell type-specific biological processes activated independently of perturbation status (SFig. 5 and Supplementary Data 1).

### Large-scale investigation of biological responses to drug treatments in cell lines

**Fig. 2:**
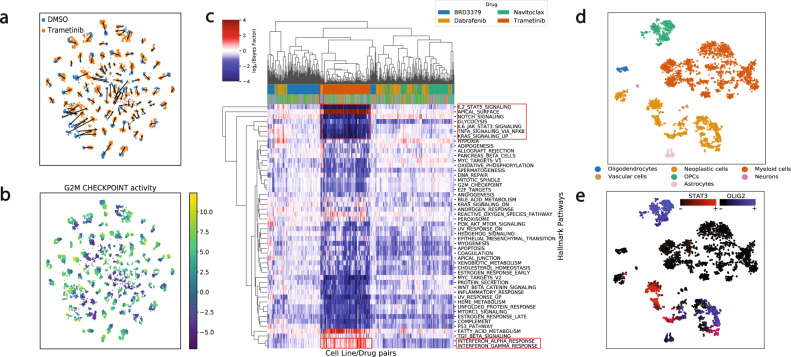
The flexibility in the latent space specification sheds light on the activity of core cellular processes and transcription factors. **a** tSNE embedding of the latent space of VEGA for the MIX-Seq data^21^. The color indicates the treatment condition, and the arrow indicates the median shift in coordinates of each cell line between the two conditions. **b** Inferred G2M checkpoint activity of each cells, showing a decreased activity in the treated condition, as expected from the MoA of Trametinib. **c** Heatmap with hierarchical clustering showing the average log_e_(Bayes Factor) of each pathway for each cell line/drug treatment pair (test between DMSO and treatment condition). Each row corresponds to a hallmark gene set and each column to a different cell line/drug pair. The first row of color indicates the drug, and the second row of color indicates the tissue identity (Tissue legend available in SFig. 2). Highlighted cell lines correspond to BRAF-mutant melanoma. Highlighted activities correspond to Trametinib-specific responses. **d** tSNE embedding of the latent space of the model for the glioblastoma dataset^29^, colored by cell type or **e** Inferred activity of the master regulators STAT3 and OLIG2.

Next, we investigated whether VEGA could detect patterns of drug responses in large-scale experiments over cancer cell lines, such as the data introduced in recent experimental protocols like MIX-Seq^21^. To this end, we gathered single-cell data for 97 cancer cell lines under five different conditions: 24 h DMSO treatment (control), 24 h Trametinib treatment (MEK inhibitor), 24 h Dabrafenib treatment (Mutated BRAF inhibitor), 24 h Navitoclax treatment (Bcl-2 inhibitor), and 24 h BRD3379 treatment (tool compound with unknown mode of action, MoA) (Methods). We trained one model for each different drug treatment (four models in total) by combining the drug treatment dataset and the control group (DMSO dataset), initializing the GMVs of VEGA with the hallmark gene sets from MSigDB^22^ to focus on core cellular processes. Overall, each model was able to separate cell lines and treatment conditions in the GMV space (Fig. 2a, and SFig. 6). For Trametinib notably, the important change in G2M checkpoint GMV activity (decrease in the treated condition) agrees with the expected MoA of a MEK inhibitor^23,24^ (Fig. 2b). Next, we sought to investigate whether we could recapitulate the pattern of biological responses between control and treated conditions for each cell line/drug treatment pair. For each pair, we computed GMV BFs to approximate differential pathway activities between the two conditions. The resulting heatmap can be used to understand and interpret patterns of response over all experimental conditions (Fig. 2c). As found when visually investigating the low dimensional embedding of each dataset (Fig. 2a and SFig. 6a–c), Trametinib resulted in the strongest transcriptional response of all studied drugs. Notably, the Trametinib-specific interferon-*α* and interferon-*γ* response was correctly recapitulated in VEGA’s latent space, consistent with previous experimental work^25^ and the findings reported by the original MIX-Seq authors^21^. Furthermore, we found that Dabrafenib-treated BRAF-mutant melanoma cell lines exhibited larger ∣log_e_(BF)∣ than other Dabrafenib-treated cell lines (average ∣log_e_(BF)∣ of 0.763 vs 0.668 for other cell lines), clustering with the Trametinib-treated cell lines as reported in the MIX-Seq study (Fig. 2c and SFig. 6d). Overall, the results presented here agree with the previous gene set analysis results on this dataset, and demonstrate VEGA’s GMVs can recapitulate patterns of drug response in large-scale experiments.

### Gene regulatory analysis of glioblastoma reveals stratification of neoplastic cells

As previously mentioned, one of VEGA’s strengths is the flexibility in the specification of the GMV connectivity, as any gene module can be used in the decoder. Transcription factors often exert tight regulation of gene expression in many biological contexts^26^. Analyzing the activity of transcriptional regulators is important in understanding biological states like cell types or diseases, as dysregulation in their activity can have a dramatic impact on gene expression programs and phenotypes^27,28^. To this end, we investigated whether using master transcriptional regulators as the GMVs could help understand the underlying GRNs in the context of a single-cell glioblastoma (GBM) dataset^29^. We used the GBM ARACNe^16^ network reported in Carro et al.^28^ to guide the structural design of our model. Specifically, VEGA’s GMVs were set to the reported transcription factors and the connectivity matrix **M**, defining the GMVs decoding architecture, was created from the set of predicted target genes of each transcription factor. After training, we found that the pre-annotated cell types were well-separated in the latent space (Fig. 2d). We examined the activity of STAT3 and OLIG2, two well-known master regulators of the mesenchymal (MES) and proneural (PN) GBM subtypes, respectively. We confirmed that their GMV activity was largely anticorrelated in neoplastic cells (Fig. 2e). Additionally, OLIG2, a known master regulator of oligodendrocytes differentiation^30^, was inferred as activated in oligodendrocyte precursor cells (OPCs). These results demonstrate that VEGA is able to home-in on the relevant transcriptional regulators when the decoder wiring is extended to model known factor-to-target relationships.

### Combining cell type and cellular state representations refines cortical organoid development analysis

A great challenge of modern cellular biology is to identify and define cell types and cellular states, at the level of individual cells, in order to systematically study homeostasis and disease development under a common vocabulary. In a typical single-cell study, a few “marker sets” will be known, each containing a list of genes having expected expression patterns for some of the cell types of interest. Leveraging such marker sets often provides clues and helps orient data analysis. We asked whether the information recorded in such marker sets could be used in VEGA to produce a disentangled representation of cell types and cellular states. To this end, we added a GMV *z*_*t*_, with appropriate entries in **M**, for each latent cell type *t* in addition to the Reactome pathway GMVs already in VEGA’s model.

**Fig. 3:**
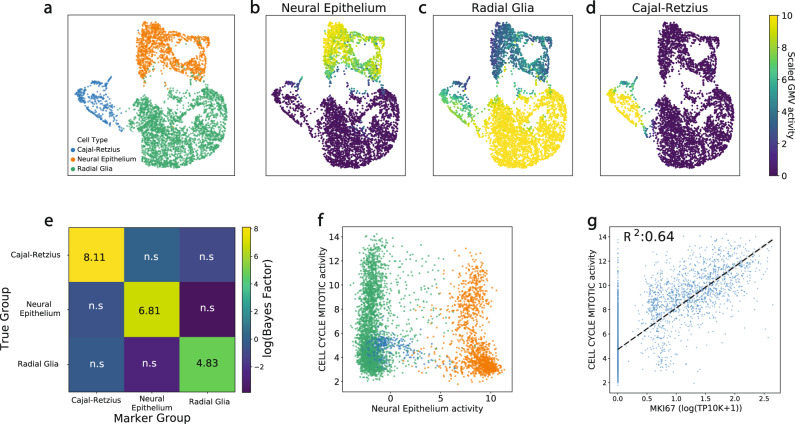
Disentangling cellular states and cell types in the early development of cortical organoids. **a** UMAP embedding of the latent space of our model for the week 2 cortical organoid dataset^31^. The cell type annotation corresponds to the original paper annotation. **b**, **c**, **d** The inferred activity of each cell type GMVs (as defined by marker genes) correctly identifies the three main subpopulations of cells. **e** One-vs-rest differential GMV analysis of each cell type population provides a statistical significance for each cell type signature. The significance threshold for positive enrichment was set to log_e_(BF)> 3. **f** Identification of dividing and quiescent subpopulations of neural progenitors using pathway and cell-type activity projection. **g** CELL_CYCLE_MITOTIC pathway activity correctly identifies dividing cells as reported by its correlation with MKI67 gene expression (an external canonical marker of dividing cells).

We applied VEGA to a dataset of cells assayed during the early development of cortical organoids from Field et al.^31^, including all of the major cell types defined in the study as GMVs (Fig. 3a). After training, we found that the activity of each marker set GMV was able to correctly segregate its corresponding cell type as annotated by the original authors (Fig. 3b–d). Moreover, in a one-vs-rest differential GMV analysis setting for each cell type population, the activity of the corresponding marker set GMV showed significant enrichment (∣log_e_(BF)∣ > 3), which suggests using GMV BFs could help annotate the cell types of unknown clusters (Fig. 3e). We further noted that the most differentially activated GMVs were coherent in the context of early brain development (SFig. 7 and Supplementary Data 2). To study whether VEGA could separate cell type identity from cellular states such as dividing vs quiescent cell populations, we projected the dataset into two components: (1) the cell type GMV representing the neural epithelium marker set (a type of early brain progenitor) and (2) the cell state GMV representing the cell cycle mitotic pathway activity (Fig. 3f). As discussed previously, the activity of the neural epithelium GMV separated the neural epithelium cells from the rest of the dataset, while the activity of the cell cycle mitotic pathway GMV separated quiescent from actively dividing cells in the two progenitors populations (radial glia cells and neural epithelium). To validate that the cells identified as dividing were proliferating, we studied the correlation between the cell cycle mitotic pathway GMV activity and the expression of the MKI67 gene, a canonical marker of proliferation (external validator not present in the cell cycle mitotic pathway set) (Fig. 3g). Overall, the expression of MKI67 correlates well with the inferred activity of the cell cycle mitotic pathway GMV (*R*^2^ = 0.64). Together, these results demonstrate VEGA’s potential use to jointly infer cell type and state for different populations of cells, as combining different sources of information (pathways, master regulators, and cell type markers) in the latent space can shed light on different aspects of the identity of a single-cell.

### Generalization of the inference process to out-of-sample data

We next asked whether VEGA could generalize to correctly infer an interpretable latent representation of data unseen at the time of training (out-of-sample data). To this end, we evaluated VEGA in two settings. In the first case, we measured the biological generalization of VEGA’s inference by holding out (cell type, condition) pairs during training. Specifically, we investigated whether the inferred GMV activities for held-out cells were conveying the same biological information as to when this population is seen at the time of training. To this end, we removed one cell type of the stimulated condition during training, and then inferred the GMV activities for that held-out population (out-of-sample) and compared them to the GMV activities learned from the fully trained model. The experiment was conducted using the Kang et al.^17^ PBMC dataset. In the second case, we estimated the “technical generalization” of VEGA’s inference by training on one dataset (study A) and then evaluating on a second dataset (study B) that contains only control cells. We used the Kang et al.^17^ PBMC dataset as study A and the Zheng et al.^32^ dataset as study B.

**Fig. 4:**
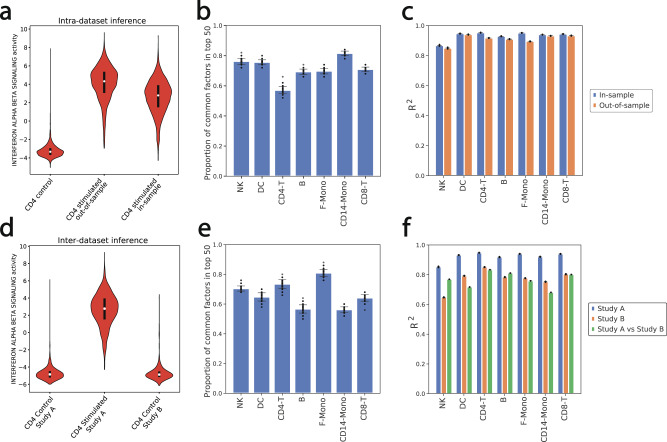
Generalization of VEGA architecture to out-of-sample data. **a** Violin plot (*n* = 10,000 randomly sampled cells per condition) representing the distribution of the interferon-*α*/*β* pathway activity in control CD4-T cells, stimulated CD4 T cells unseen at the time of training (out-of-sample), and stimulated CD4-T cells when included in the training procedure (in-sample). Boxes inside the violins represent the median of the distribution bounded by the first and third quartile. Violin limits correspond to data extrema. **b** Proportion of overlap in the top 50 differentially activated GMVs in the in-sample and out-of-sample settings with stimulated vs control differential procedures for the seven main cell types in the study. Data were presented as mean values ± standard deviation over 100 random sampling. **c** *R*^2^ between the mean expression of real and reconstructed cells in the in-sample and out-of-sample settings for the seven main cell types of the study. Data were presented as mean values ± standard deviation over 100 random samplings. **d** Violin plot (*n* = 2000 randomly sampled cells per condition) of distribution of the interferon-*α*/*β* pathway activity in control CD4-T cells of study A (Kang et al.^17^), stimulated CD4-T cells of study A and control CD4-T cells of study B (Zheng et al.^32^). Boxes inside the violins represent the median of the distribution bounded by the first and third quartile. Violin limits correspond to data extrema. **e** Proportion of overlap in the top 50 differentially activated GMVs of each study with one-vs-rest differential procedures for the control cells of the seven main PBMC cell types. Data were presented as mean values ± standard deviation over 100 random samplings. **f** *R*^2^ between the mean expression of real and reconstructed cells of study A (Study A), mean expression of real and reconstructed cells of study B (Study B), and mean expression of real cells of study A and real cells of study B (Study A vs Study B). Data were presented as mean values ± standard deviation over 100 random samplings.

For the biological generalization test, we first checked that the distribution of the interferon-*α*/*β* signaling pathway GMV activity in the out-of-sample stimulated CD4 T cells matched the inferred activity in the in-sample CD4 T cells (Fig. 4a). To perform a more systematic comparison of the inferred latent space between out-of-sample and in-sample cells, we used the differential BF procedure (Methods) between (1) stimulated in-sample cells and control cells for a given cell type (model trained with the whole dataset) and (2) stimulated out-of-sample cells and control cells for the same cell type (model trained with one cell type/condition pair left out), and checked the amount of overlaps in the top 50 differentially activated GMVs (Fig. 4b). The results suggested consistency between the in-sample and out-of-sample differentially activated GMVs, with an average 72% overlap. To further evaluate the capacity of data reconstruction, we measured the *R*^2^ between the original and decoded data in the in-sample and out-of-sample settings (Fig. 4c). We found that the *R*^2^ decreases only marginally in the out-of-sample setting, confirming the ability of the model to generalize to unseen data produced in a similar experimental setting.

For the technical generalization test, we again checked that the interferon-*α*/*β* signaling pathway GMV activity distribution of study B encoded control CD4 T cells matched that of study A control CD4 T cells (Fig. 4d). We also investigated whether the top 50 differential GMVs of each cell type in a “one-vs-rest” differential setting for the control cells of study A overlapped with a similar procedure performed on the control cells of study B (Fig. 4e). We found that on average 67% of the top 50 differential GMVs for study A overlap with those of study B, showing that the model can generalize across studies unseen at the time of training. We then asked whether the model can use the inferred latent space to accurately reconstruct the original expression profiles of both studies. We found that the *R*^2^ between original and reconstructed cells of study B, although lower than those for study A, improves upon the baseline correlation between the expression profiles of study A vs study B for most of the cell types (Fig. 4f).

## Discussion

In this study, we introduced VEGA, a novel VAE architecture with a decoder inspired by known biology to infer the activity of various gene modules at the level of individual cells. By encoding single-cell transcriptomics data into an interpretable latent space specified a priori, our method provides a fast and efficient way of analyzing the activity of various biological abstractions in different contexts. In contrast, previous approaches used a posteriori interpretations of the latent variables to infer modules. VEGA’s flexibility in the specification of the latent space paves the way for analyzing the activity of biological modules such as pathways, transcriptional regulators, and cell type-specific modules. We illustrated how VEGA could be used to simultaneously investigate both cell type and cell state of cell subpopulations, in both control and experimentally perturbed conditions. Additionally, the weights of decoder connections provide direct interpretability of the relationship between the latent variables and the original features. For example, the decoder’s weights could be used to contrast interaction confidence in inferred GRNs or to rank genes by their importance in a certain biological module in a data-driven way. We further note that it was possible to modify VEGA’s architecture, following the same rationale as widely-used scVI^9^ and linear scVI^13^, such that it could handle count data in place of normalized expression profiles (SFig. 8).

The clear limitations of the current architecture resides in the sparse, single-layer decoder of the model. In fact, such an architectural design prevents the further improvement of generalizability and robustness. As a consequence, the generative capacity of VEGA is limited. For example, while VEGA theoretically could be used for interpretable response prediction using latent vector arithmetics in a similar fashion to scGen^10^, VEGA’s limited generative capacity sacrifices predictive performance for biological interpretability of the latent space. We believe advanced insights in network biology, e.g., multi-layer GRNs that can describe regulatory machinery more comprehensively, could alleviate these limitations. This would open the possibility to perform targeted, in-silico activation, and repression of biological programs on specific cell populations to study its effect on development or disease progression. On the other hand, hard-coded connections of the linear decoder do not leave any room for correcting prior knowledge about gene modules when the context requires it, as is the case in other latent variable models such as f-scLVM^12^. In fact, prior biological knowledge obtained from existing databases like MSigDB can be incomplete or not context-specific, as additional unannotated genes can play an important role in certain gene modules. In parallel to our work on VEGA, Rybakov et al.^33^ introduced a regularization procedure to incorporate prior knowledge from gene annotation databases via a penalty term on the weights of the linear decoder. We demonstrated that VEGA performs comparatively to their interpretable autoencoder (SFig. 9), and that their approach is complementary to the unique attributes of VEGA and can be used to recover missing gene-GMV links in a data-driven fashion (SFig. 10).

In summary, we found VEGA useful for understanding the response of specific cell type populations to different perturbations, providing interpretable insights on biological module activity. The variational aspect of VEGA provides an advantage for addressing queries about samples, or sample groups, that are not possible with a regular AE. We illustrated how the latent multivariate Gaussian distribution of the VAE, which approximates the posterior probability of every GMV, enables a new kind of differential test to be performed. The BF reflects the likelihood of how active a gene module is in one condition compared to another, providing a straightforward method to perform differential activity analysis using the RNA-Seq data similar to the approach described by Lopez et al.^9^. Other types of queries are possible, for example, to automate the annotation of unsupervised clusters or modules that dynamically change across the branches of an inferred cellular trajectory. We envision VEGA could also be useful to prioritize drugs based on pathway expression in cancer, as studying the response of specific cell populations may inform drug sensitivity and resistance. Integrating drug response prediction models with such explanatory models could benefit designing novel therapeutic strategies.

## Methods

### The VEGA architecture

VEGA is a deep generative VAE that aims at maximizing the likelihood of a single-cell dataset *X* under a generative process^7,10^ described as:

1 $$p(X| \theta )=\int p(X| Z,\theta )p(Z| \theta )dZ,$$

with *θ* being the learnable parameters of a neural network. VEGA uses a set of latent variables *Z* that explicitly represent sets of genes (gene modules), such as pathways, GRNs, or cell type marker sets. To enforce the VAE to interpret a dataset from the viewpoint of a set of gene modules, VEGA’s decoder part is made up of a single, masked, linear layer. Specifically, the connection of this layer, between latent node *z*^(*j*)^ and gene features, are specified using a binary mask **M** in which *M*_*i*,*j*_ is true if gene *i* is a member of gene module *j* and false otherwise. We refer to each latent variable *z*^(*j*)^ as a GMV since each provides a view of the data constrained to the subset of genes for a distinct gene module *j*. During training, gradients associated with masked (false) weights are “zeroed out” such that backpropagation only applies to weights originating from a user-supplied given gene set. Additionally, the weights of the decoder are constrained to be nonnegative (*w* ≥ 0) to maintain interpretability as to the directionality of gene module activity.

Having explicitly specified the connections between genes and latent variables in the decoder of VEGA (generative part), we incentivize that the latent space represents a biological module activity interpretation of the data. We choose to model the GMVs as a multivariate normal distribution, parametrized by our inference network with learnable parameters *ϕ* As such, the distribution of the Z latent variables can be expressed as:

2 $$q(Z| X,\phi )={{{{{{{\mathcal{N}}}}}}}}({\mu }_{\phi }(X),{{{\Sigma }}}_{\phi }(X))$$

This choice of variational distribution is common and has proven to work well in previous single-cell studies^9,10^. Following similar standard VAE implementations^7,10^, the objective to be maximized during training is the evidence of lower bound (ELBO):

3 $${{{{{{{\mathcal{L}}}}}}}}(X)={{{{{\rm{E}}}}}}_{q(Z| X,\phi )}\left[{{{{{{\mathrm{log}}}}}}}\,p(X| Z,\theta )\right]-KL(q(Z| X,\phi )| | p(Z| \theta ))$$

where the expectation over the variational distribution can be approximated using Monte Carlo integration over a minibatch of data, and the Kullblack–Leibler divergence term has a closed-form solution as we set the prior to:

4 $$p(Z| \theta ) \sim {{{{{{{\mathcal{N}}}}}}}}({{{{{{{\bf{0}}}}}}}},{{{{{{{\bf{I}}}}}}}})$$

The reparametrization trick^7^ is used when sampling VEGA’s variational distribution to allow standard backpropagation to be applied when training the model.

To retain information of genes that are not present in our pre-annotated biological networks, we add additional fully connected nodes to the latent space of our model. This has two effects: (1) it allows VEGA to model the expression of unannotated genes, which could be crucial for a good reconstruction of the data during training, and (2) it can help capture additional variance of the data that is unexplained by the provided gene modules, considerably improving the training of the model. The number of additional fully connected nodes can be determined based on a trade-off between model performances and the loss of information encoded by pre-annotated GMV nodes. As a rule of thumb, we recommend picking 16 or fewer extra FC nodes to preserve the biological signals encoded by GMV nodes (SFig. 11).

Additionally, the diagonal covariance prior used in the latent space modeling discourages GMVs from being correlated. Thus, the VAE may be forced to choose an arbitrary gene set among many equally informative but overlapping sets and could fail to reveal a key annotation. To address this issue, we add a dropout layer to the latent space of the model. This has been shown to force the VAE to preserve redundancy between latent variables^34^, which is applicable when the gene annotation database used to initialize VEGA’s latent space contains overlapping gene sets (SFig. 12).

Finally, batch information or other categorical covariates can be encoded via extra nodes in the latent space, conditioning the generative process of VEGA on this additional covariate information (SFig. 2).

### Measuring differential GMVs activity of the latent space with Bayes Factor (BF)

The difference in the activity of genes and/or pathways is often of interest when contrasting two different groups of cells. To this end, we draw inspiration from the Bayesian differential gene expression procedure introduced in Lopez et al.^9^ and propose a similar differential GMV analysis procedure. We follow a similar notation as Lopez et al. For a given GMV *k*, a pair of cells (*x*_*a*_, *x*_*b*_) and their respective group ID (*s*_*a*_, *s*_*b*_) (e.g., two different treatment conditions), our two mutually exclusive hypotheses are:

5 $${{{{{{{{\mathcal{H}}}}}}}}}_{0}^{k}:={{{{{\rm{E}}}}}}_{s}\left[{z}_{a}^{k}\right] \, > \, {{{{{\rm{E}}}}}}_{s}\left[{z}_{b}^{k}\right]\ {{{{{{{\rm{vs.}}}}}}}}\ {{{{{{{{\mathcal{H}}}}}}}}}_{1}^{k}:={{{{{\rm{E}}}}}}_{s}\left[{z}_{a}^{k}\right]\,\le\, {{{{{\rm{E}}}}}}_{s}\left[{z}_{b}^{k}\right]$$

This can intuitively be seen as testing whether a cell has a higher mean GMV activation than another, the expectation representing empirical frequency. We evaluate the most probable hypothesis by studying the log-Bayes factor *K* defined as:

6 $$K={{{{{{{\mathrm{log}}}}}}}\,}_{e}\frac{p({{{{{{{{\mathcal{H}}}}}}}}}_{0}^{k}| {x}_{a},{x}_{b})}{p({{{{{{{{\mathcal{H}}}}}}}}}_{1}^{k}| {x}_{a},{x}_{b})}$$

Here, the sign of *K* tells us which hypothesis is more likely, and the magnitude of *K* encodes a significance level. Having access to the conditional posterior distribution q(Z∣X) over the GMVs activation (the encoding part of VEGA), we can approximate each hypothesis’ probability distribution as:

7 $$p\left({{{{{{{{\mathcal{H}}}}}}}}}_{0}^{k}| {x}_{a},{x}_{b}\right)\approx \mathop{\sum}\limits_{s}p\left(s\right)\ \ \mathop{{{{\iint}}}}\limits_{{{{{{{{\rm{sup.}}}}}}}}({z}_{a}),\ {{{{{{{\rm{sup.}}}}}}}}({z}_{b})}p\left({z}_{a}^{k} \, > \, {z}_{b}^{k}\right)dq\left({z}_{a}^{k}| {x}_{a}\right)dq\left({z}_{b}^{k}| {x}_{b}\right)$$

where *p*(*s*) is the relative abundance of cells in group *s*, and the integrals are approximated with direct Monte Carlo sampling.

Similarly to Lopez et al.^9^, assuming cells are independent, we can compute the average Bayes factor across many cell pairs randomly sampled from each group respectively. This helps us decide whether a GMV is activated at a higher frequency in one group or the other. Through the paper, we consider GMVs to be significantly differentially activated if the absolute value of *K* is greater than 3 (equivalent to an odds ratio of ≈20)^9,20^.

### Datasets and preprocessing

#### Kang et al. dataset

The Kang et al.^17^ dataset consisted of two groups of PBMCs, one control and one stimulated with interferon-*β*. We chose to use the same preprocessing steps as described by scGen authors^10^, using the Scanpy package^35^. Briefly, cells were annotated using the maximum correlation to one of the eight original cell type clusters identified, using an average of the top 20 cluster genes. Megakaryocytes were removed due to uncertainty about their annotation. Then data were filtered to remove cells with less than 500 genes expressed and genes expressed in five or less cells, using the scanpy.pp.filter_genes()and scanpy.pp.filter_cells() functions. Count per cells were then normalized and log-transformed using the scanpy.pp.normalize_per_cell() and scanpy.pp.log1p() functions, and we selected the top 6998 highly variable genes with scanpy.pp.highly_variable_genes(), resulting in a final dataset of 18,868 cells. Raw data is available at GSE96583. We used the same preprocessing functions for the rest of the datasets unless specified otherwise.

#### Zheng et al. dataset

The Zheng et al.^32^ dataset consists of 3K PBMCs from a healthy donor. After filtering the cells, the count per cells were normalized and log-transformed. We then subset the genes to use the same 6998 genes of the Kang et al. PBMC dataset. The final dataset has 2623 cells and 6998 genes. Raw data are available at https://support.10xgenomics.com/single-cell-gene-expression/datasets/1.1.0/pbmc3k.

#### MIX-seq dataset

The MIX-seq^21^ datasets were obtained from https://figshare.com/s/139f64b495dea9d88c70, and we used the data from experiment 3 to have enough cells to carry a smooth training of our model. For the five available datasets (97 cell lines treated with respectively DMSO, Trametinib, Dabrafenib, Navitoclax, and BRD3379), we removed cells with 200 or less expressed genes, and genes expressed in less than three cells. We then normalized the number of counts per cell, and log-transformed the data. Finally, each dataset that was a drug treatment experiment was combined with a copy of the control dataset (DMSO treatment), and we extracted the top 5000 highly variable genes. This resulted in final datasets of size (16,732 cells and 4999 genes) for the Trametinib+DMSO data, (16,942 cells and 5000 genes) for the Dabrafenib+DMSO data, (14,507 cells and 5000 genes) for the Navitoclax+DMSO data, and (15,304 cells and 5000 genes) for the BRD3379+DMSO data.

#### Darmanis et al. dataset

The raw GBM data from Darmanis et al.^29^ were obtained from http://www.gbmseq.org/ and preprocessed as followed: we removed cells with 200 or less expressed genes, and genes expressed in three or less cells. Count per cells were normalized and data were then log-transformed. Finally, we restricted the transcriptome to the top 6999 highly variable genes. The final dataset had a total of 3566 cells. Raw data is available at GSE84465.

#### Field et al. dataset

The cortical organoid data from Field et al.^31^ was processed similarly to the GBM dataset. After normalization and highly variable genes selection, the dataset had a total of 4378 cells, with 6999 genes. Raw data is available at GSE106245.

#### Shekhar et al. dataset

The mouse retina dataset from Shekhar et al.^36^ was processed as described (see https://github.com/broadinstitute/BipolarCell2016). Briefly, we removed cells with more than 10% mitochondrial transcripts. Then, cells with less than 500 genes were removed, and genes expressed in less than 30 cells and with less than 60 transcripts across all cells were removed. To be able to use human versions of gene modules from the Reactome database, we performed one-to-one ortholog mapping of mouse transcripts to human transcripts using BioMart from the Ensembl project^37^. Genes without human orthologs were removed. We saved a version of the dataset with the raw count data for the selected genes/cells, and further processed the data by normalizing and log-transforming the libraries. Finally, we restricted the transcriptome to the top 4000 highly variable genes. The same highly variable genes were used to subset the raw QC count matrix. The final datasets (for both count and log-normalized versions) had a total of 27,499 cells, coming from two technical batches. We used the annotation with 15 cell types from the original authors. Raw data is available at GSE81904.

### Choice of gene annotations for the latent space of VEGA

When initializing the latent space of our model, we chose to use pre-annotated gene sets from the Molecular Signature Database (MSigDB, at https://www.gsea-msigdb.org/gsea/msigdb/collections.jsp#C2)^14^. In particular, we chose to use the hallmark gene sets annotation (50 gene sets) or the Reactome database (674 gene sets). Reactome was used for the stimulated PBMCs analysis, and MSigDB’s Hallmark gene sets were used in the MIX-Seq analysis part of this study. For the gene regulatory network analysis of GBM cells, we derived an ARACNe^16,38^ network from bulk RNA-Seq samples of GBM. Specifically, this network was obtained from a previously published paper^39^ and repurposed for the study of GBM single-cell transcriptomics profiles.

For the cell type marker genes in the cortical organoid analysis, we contacted the authors to obtain relevant genes used in annotating those cell types. The GMT file including these marker genes can be found along with the reproducibility code at https://github.com/LucasESBS/vega-reproducibility.

### Dimensionality reduction for visualization

For visualizing datasets, we used the UMAP algorithm^40^ as implemented in the Scanpy^35^ python package, using scanpy.pp.neighbors() for the k-NN computation with n_neighbors=15, and scanpy.tl.umap() for the actual dimensionality reduction. We used default parameters except for the min_dist parameter that we set to 0.5. We also used tSNE^41^ implemented as sklearn.manifold.TSNE() in the sklearn python package^42^, with default parameters.

### Comparison with GSEA

We ran Gene Set Enrichment Analysis https://www.zotero.org/google-docs/?grfpAv^14^ (GSEA) using the prerank function from the gseapy package in Python. Briefly, we calculated differential expression scores for each gene between the control and treatment group using a Wilcoxon rank-sum test, as implemented in the scanpy.tl.rank_genes_groups() functionality of the Scanpy package https://www.zotero.org/google-docs/?fKytT7^35^. We ranked genes according to their test statistics, and ran GSEA using the gseapy package function gseapy.prerank() with the following settings: a minimum gene set size min_size=5, a maximum gene set size max_size=1000, and a number of permutations permutation_num=1000. We ranked gene sets according to their FDR and considered significant hits when FDR ≤0.05. When the FDR returned by GSEA was equal to 0, we replaced it with 1e-5 (to avoid math error when taking the logarithm).

### Batch correction comparison

To assess batch information integration in VEGA’s latent space, we compared the average silhouette scores on batch labels from the Shekhar et al. retina dataset of (1) PCA with 50 principal components (computed using scanpy.tl.pca() function), (2) linear scVI^13^ as implemented in the scvi-tools package ran on the count version of the dataset with following parameters: AnnData object setup with batch_key=Batch, model initialized with n_hidden=800, n_layers=2, dropout_rate=0.2, n_latent=677, training performed with max_epochs=300, early_stopping=True, lr=5e-4, train_size=0.8, early_stopping_patience=20, and (3) VEGA with following parameters: AnnData object setup with batch_key=atch, model initialized using the REACTOME pathway database with three extra FC nodes to initialize the latent space and the same training hyperparameters as linear scVI.

#### Evaluation metrics

Silhouette scores were calculated to evaluate the separation of cell types and states in the latent space of our model. We used Euclidean distance in the latent space to compute the silhouette coefficient of each cell *i* defined as :

8 $$s(i)=\frac{b(i)-a(i)}{\max \{a(i),b(i)\}}$$

where *a*(*i*) and *b*(*i*) are respectively the mean intra-cluster distance and the mean nearest-cluster distance for cell *i*. We used either the stimulation or cell type labels from Kang et al.^17^ to assess the biological relevance of the latent space of our model. The sklearn package^17^silhouette_score() implementation was used for computation. For computing correlations throughout the paper, we used the function numpy.corrcoef() from the Numpy package^43^.

### Reporting summary

Further information on research design is available in the Nature Research Reporting Summary linked to this article.

## Supplementary information

Supplementary Information

Description of Additional Supplementary Files

Supplementary Data 1

Supplementary Data 2

Reporting Summary

## Data Availability

All of the datasets analyzed in this manuscript are publicly available. Please see the section Datasets and preprocessing of Methods for details. These datasets are also downloadable at https://github.com/LucasESBS/vega-reproducibility.

## Code availability

The package and API for VEGA is available at https://github.com/LucasESBS/vega/tree/vega_dev^44^. The code and data to reproduce the results of this manuscript is available at https://github.com/LucasESBS/vega-reproducibility.
