# Supplementary material for: VEGA is an interpretable generative model for inferring biological network activity in single-cell transcriptomics
Source: Nat Commun. 2021 Sep 28;12:5684. doi: 10.1038/s41467-021-26017-0 (PMC8478947; doi:10.1038/s41467-021-26017-0)
Supplement: Supplementary file 1 — Supplementary Information [file 41467_2021_26017_MOESM1_ESM.pdf]

Supplementary Information

VEGA architecture and training procedure

Name	Operation	NoF	Dropout	BN	Activation	Input
input	-	input_dim	No	No	-	-
FC-1	FC	800	Yes	Yes	ReLU	input
FC-2	FC	800	Yes	Yes	ReLU	FC-1
mean	FC	latent_dim	Yes	No	Linear	FC-2
logvar	FC	latent_dim	Yes	No	Linear	FC-2
z-sample	FC	latent_dim	No	No	Linear	[mean,logvar]
SD	SC	input_dim	No	No	Linear	z-sample

Deep encoder/ sparse decoder (VEGA) architecture

NoF: Number of Features. FC: fully connected. BN: batch normalization. ReLU: rectified linear unit. SD: sparse decoder. SC: Sparsely connected

Unless specified otherwise in the Method section, we used the following hyperparameters for VEGA:
For training, we used a learning rate of 1e-4 with the Adam optimizer.
The KL divergence was weighted with a factor β=0.0001.
The dropout rate was set to 0.5 for the encoder and latent layers, except in the following case: for the batch correction experiment, encoder dropout rate was set to 0.2 and latent dropout rate was set to 0; for the dropout experiment in SFig.10, encoder dropout rate was set to 0.2 and latent dropout rate was set to either 0 or 0.5.
The batch size was set to 128 and the model was trained for a maximum of 500 epochs. For the GBM dataset from Darmanis et al., batch size was set to 64.
To avoid overfitting, we used Early stopping: we stopped the training procedure when the training loss or test loss stopped increasing for 10 consecutive epochs.
Whenever we trained a model, we used a 80%/20% train-test split.

The latent dimension was defined by the number of biological modules used for the analysis, plus the number of additional fully connected nodes used to capture additional variance. The following dimensions were used:
Kang et al. dataset: 674 reactome pathways + 1 FC node = 675
MIX-seq dataset: 50 hallmark pathways + 5 FC nodes = 55
Darmanis et al. dataset: 6056 transcription factors + 5 FC nodes = 6061

Field et al. dataset: 674 reactome pathways + 7 cell type sets + 1 FC node = 682
Shekhar et al. dataset: 674 reactome pathways + 3 FC nodes = 677

Alternative architectures for SFig.1:

Name	Operation	NoF	Dropout	BN	Activation	Input
input	-	input_dim	No	No	-	-
sparse mean	SC	latent_dim	0.5	Yes	ReLU	input
sparse logvar	SC	latent_dim	0.5	Yes	ReLU	input
z-sample	FC	latent_dim	No	No	Linear	[sparse mean, sparse logvar]
SD	SC	input_dim	No	No	Linear	z-sample

Sparse encoder/ sparse decoder architecture

Name	Operation	NoF	Dropout	BN	Activation	Input
input	-	input_dim	No	No	-	-
sparse mean	SC	latent_dim	0.5	No	Linear	input
sparse logvar	SC	latent_dim	0.5	No	Linear	input
z-sample	FC	latent_dim	No	No	Linear	[sparse mean, sparse logvar]
FC-1	FC	800	0.5	Yes	ReLU	z-sample
FC-2	FC	800	0.5	Yes	ReLU	FC-1
FC-3	FC	input_dim	No	No	Linear	FC-2

Sparse encoder/ deep decoder architecture

Name	Operation	NoF	Dropout	BN	Activation	Input
input	-	input_dim	No	No	-	-
FC-1	FC	800	0.5	Yes	ReLU	input

FC-2	FC	800	0.5	Yes	ReLU	FC-1
mean	FC	latent_dim	0.5	No	Linear	FC-2
logvar	FC	latent_dim	0.5	No	Linear	FC-2
z-sample	FC	latent_dim	No	No	Linear	[mean,logvar]
FC-1	FC	800	0.5	Yes	ReLU	z-sample
FC-2	FC	800	0.5	Yes	ReLU	FC-1
FC-3	FC	input_dim	No	No	Linear	FC-2

Deep encoder/ deep decoder architecture (standard VAE, not interpretable)

Training for SFig.1 was done similarly to previously described, except for the early stopping patience which was increased to 25 epochs to ensure good convergence was reached for all alternative architectures.

SFig.1: Alternative architectures for the interpretable VAE and performance. (A) We evaluated 3 different architectures for our VAE which all provide interpretability through explicit link between original features and latent variables, as well as a standard VAE architecture for reference: (1) A sparse encoder and sparse decoder architecture, (2) a deep encoder and sparse decoder architecture, (3) a sparse encoder and deep decoder architecture and (4) a standard VAE architecture (not interpretable). (B) Average silhouette score in the gene expression space (baseline control) and in the latent space of each architecture over 10-fold cross validation (data are presented as mean values +/- standard deviation, n=10 independent models) for separating the two experimental conditions (control vs. stimulated, blue), the cell types of the control condition (orange) and the cell types of the stimulated condition (green). (C) Best validation loss (MSE + KL divergence) for each architecture over 10-fold cross validation when training with early stopping (n=10 independent models). Middle line corresponds to the median loss, the edges of the boxes correspond to lower and upper quartiles, and the whiskers denote the data range (extremes). Evaluation was done on the Kang et al.[1] dataset.

Supplementary Note 1:
To decide which interpretable architecture to use for VEGA, we evaluated three possible ways of connecting latent variables to gene features, through the encoder or the decoder. For reference, we also included a standard VAE architecture, which doesn’t provide direct interpretability. We evaluated two aspects: the ability to recapitulate the biological signal in the latent space and separate conditions / cell types in the Kang et al. dataset using silhouette scores (see METHODS), including a baseline control using the original gene expression space, and the performance in terms of VAE objective (see

METHODS). While the sparse decoder/deep decoder architecture minimizes the loss function better than other architectures (as expected due to its higher generative capacity), the deep encoder/sparse decoder architecture recapitulates the biology of the dataset better over its latent space (higher silhouette scores for the three aspects of the dataset), driving our choice of architecture for the rest of the paper. VEGA improves upon the baseline silhouette score from the gene expression space, suggesting that VEGA can enrich meaningful biological information.

SFig.2: Integrating batch information in VEGA modelling to correct for technical differences. (A) UMAP embedding of the top 50 PCs in the Shekhar et al.[2] Retina dataset, colored by batch label (top) and cell type (bottom). (B) UMAP embedding on linear scVI latent space colored by batch label and cell type. (C) UMAP embedding on VEGA latent space colored by batch label and cell type.

Supplementary Note 2:
Batch effect is a common source of noise in scRNA-Seq data. To improve VEGA's GMVs inference during training when cells come from different sources, we condition the sparse, linear decoder of VEGA on batch annotation when available. This is inspired by modelling of batch information in deep generative models such as scVI[3].
To confirm that VEGA was able to use batch information to improve integration of batches in its latent space, we applied it to a retina cells dataset sequenced in two different rounds of Drop-Seq[2]. We quantify the batch integration using the average silhouette width (ASW): a lower silhouette width indicates a better integration of batches in the latent space. We compare VEGA's batch integration in the GMV latent space to a simple PCA (baseline, no batch correction) and another deep generative model with linear decoder, linear scVI[4], also integrating batch information in the modelling.
We show that both linear scVI (ASW=0.0024, SFig.2B) and VEGA (ASW=0.0059, SFig.2C) outperform the uncorrected PCA (ASW=0.0380, SFig.2A), suggesting a successful integration of the batch information by both models. Linear scVI slightly outperforms VEGA in integrating datasets; however, only VEGA provides direct interpretability of the latent variables as known pathways and gene regulatory networks.

SFig.3: A trainable decoder is required to preserve biological signals in the GMV space. (A) UMAP embedding of PCA applied to the matrix ($\mathbf{X}*\mathbf{M}^T$), where **X** is the log-transformed gene expression matrix of the Kang PBMC dataset and **M** is the binary matrix encoding GMV membership (METHODS). (B) UMAP embedding of the VAE latent space when the sparse decoder is constrained to the binary matrix **M** (weights are frozen to stay 0 or 1 during training) and only the encoder is trainable. (C) UMAP embedding of VEGA latent space (trainable encoder and trainable decoder). (D) Average silhouette scores for the three evaluated models (PCA on ($\mathbf{X}*\mathbf{M}^T$), binary

decoder VAE and VEGA) on three different labels of the Kang PBMC dataset: condition, cell types in the control condition and cell types in the stimulated condition.

Supplementary Note 3:
We explored whether having a trainable decoder was necessary to preserve the biological signal in the GMV latent space. For this, we applied 3 different strategies to encode the gene expression matrix **X** of the Kang PBMC dataset in the GMV latent space: 1) $\mathbf{X}^*\mathbf{M}^T$ where **M** denotes the mask matrix, followed by PCA (SFig.3A), 2) VEGA with the sparse linear decoder constrained to be the mask matrix **M** (binary weight matrix frozen during training, SFig.3B), and 3) regular VEGA architecture, where the sparse linear decoder is trainable (SFig.3C). We studied the UMAP embedding of the latent space generated by each method (SFig.3A-C) as well as the average silhouette width (ASW) computed on different labels of the Kang PBMC dataset (condition, cell types in the control condition,cell types in the stimulated condition, SFig.3D). We show that only a trainable decoder can properly resolve the biological signal of the dataset (SFig.3C), while a simple linear encoding of the gene expression cannot separate adaptive immune cells in the different conditions (CD8T, B, CD4T, NK in SFig.3A), and a decoder constrained to a binary matrix produces a latent space with poor separation of biological entities (ASW 2-5x lower than for a model with a trainable decoder). This demonstrates that having a trainable decoder improves the capacity of the model to capture the biological signal in the Kang PBMC dataset.

SFig.4: GSEA suffers from a stronger gene set size bias than our VAE architecture. (A) Scatterplot of log10(gene set size) versus absolute value of VEGA Bayes Factors (Innate Immune stimulated vs Innate Immune control in Kang et al.). (B) Same scatterplot but using the equivalent GSEA -log10(FDR) values for the y-axis.

Supplementary Note 4:
When comparing results of our Bayesian differential activity procedure and standard GSEA preranked results, we sought to investigate whether the difference in results could be explained by a stronger dependency of GSEA results on gene set size. Indeed, GSEA seems to suffer from a strong gene set size bias while the Bayes Factor values of VEGA are more uniformly distributed.

SFig.5: One-vs-rest cell type specific differential GMV activity testing in the Kang PBMC dataset. Volcano plot presenting the $\log_e$(BF) in a one-vs-rest differential GMV activity testing procedure, where one cell type is compared to the union of the other 6 cell types in the Kang et al. PBMC dataset. The top 5 positively activated GMVs are annotated in each panel. (A) Results for CD4 T-cells, (B) Results for CD14+Monocytes,

(C) Results for B-cells, (D) Results for CD8 T-cells, (E) Results for Natural Killer cells, (F) Results for FCGRA3+ Monocytes, (G) Results for Dendritic cells.

Supplementary Note 5:
Differential gene expression testing procedures are often done in a one-vs-rest setting, where the goal is to highlight cell type-specific genes. We demonstrate that such a procedure can be performed in VEGA's latent space to highlight differentially activated pathways that are cell type specific. For example, VEGA highlights B cell-specific pathways (SIGNALING_BY_THE_B_CELL_RECEPTOR_BCR, ANTIGEN_ACTIVATES_B_CELL_RECEPTOR_LEADING_TO_GENERATION_OF_SECOND_MESSENGERS) in the B-cell vs. rest analysis (SFig.5C). Other examples include the activation of immune-related pathways in T-cells (SFig.5A,D) and monocytes (SFig.5B,F), indicating communication between related immune cell types.

SFig.6. Detailed analysis of the three other drugs of the MIX-seq dataset. (A-C) t-SNE embedding of the latent space of our model for Dabrafenib, Navitoclax and BRD3379 treatments respectively. Arrows indicate the shift of each cell line in the DMSO control (blue) and drug treated (orange) conditions. (D-F) Heatmaps of the Bayes Factor for each pathway/cell line in a "treated vs. control" differential activity setting. Rows are Hallmark gene sets and columns are individual cell lines. Red indicates activated in the treated condition while blue indicates repressed in the treated condition. Column legend: color by tissue and BRAF/KRAS mutation (tan: no mutation, brown: mutation).

Supplementary Note 6:
Here we investigate in more detail the response of cell lines to the 3 other drugs of the MIX-Seq dataset (Dabrafenib, Navitoclax, BRD3379).
In the Dabrafenib treated condition, we first notice a strongly responding cluster of several cell lines (larger Bayes Factor). When investigating the status of these cell lines, we found that strongly responding cell lines were skin cancer cell lines with BRAF mutation (SFig.6). As a mutated BRAF inhibitor, we found that it makes sense that Dabrafenib separates most of cell lines with a BRAF mutation from the rest, and induces a stronger response in those, notably decreasing the activity of cell cycle-related pathways (G2M checkpoint, mitotic spindles, etc). Those cell lines (BRAF mutant melanoma) were also found as strongly responsive in the MIX-Seq study, notably clustering with Trametinib cell lines in their analysis[5]. Other cell lines seemed to be less responsive in terms of transcriptomics changes, although some pathway activities were decreased (MYC target V1, Estrogen response late).
In the Navitoclax treated condition, we didn't find a clear clustering of cell line responses in terms of tissue or mutation status. However, we found that most cell lines had a

decreased interferon activity after 24hr treatment, which was also recently found in a multi-omics study of Navitoclax treatment on breast cancer cell lines[6]. While this study is only investigating a triple-negative breast cancer cell line, we found that most of the changes in pathway activity were recapitulated by our model.
In the BRD3379, although the MoA of the drug is unknown, we found that most down regulated pathways could be related to some cellular stress (protein secretion, UV response, IL2 signaling …). However, those results are hard to evaluate without extensive experimental validation of the effect of this drug on cell lines.

SFig.7: One-vs-rest cell type specific differential GMV activity testing in the cortical organoid dataset. Volcano plot presenting the $\log_e$(BF) in a one-vs-rest differential GMV activity testing procedure, where one cell type is compared to the union of the other 6 cell types in the cortical organoid dataset. The top 5 positively activated GMVs are annotated in each panel. (A) Results for Neural epithelium cells, (B) Results for Radial Glia cells, (C) Results for Cajal-Retzius neurons.

Supplementary Note 7:
We performed differential GMV activity testing in a one-vs-rest setting for the cell types of the cortical organoid dataset. Several highlighted pathways indicate the activation of developmental pathways and neural growth (Nuclear receptor transcription pathway in Neural epithelium, SFig.7A, GAG synthesis pathways in Radial Glia cells, Pre Notch signaling in Golgi in Cajal-Retzius neurons SFig.7C), but also metabolic stress pathways in neural stem cells (Biological oxidation in Neural epithelium, SFig.7A). This showcases the use of VEGA to highlight cell type-specific processes activated during early neural development.

SFig.8: VEGA sparse linear decoder can be adapted to model scRNA-Seq count data. (A) Modified VEGA architecture for count data modelling. One encoder is used to encode the GMV activity (*z* encoder), and a second encoder infers the latent library size scaling factor for each single-cell (*l* encoder). The decoder represents a negative binomial distribution generating over-dispersed count data, and encodes the prior knowledge from the gene annotation database. Optional batch variable is omitted in this cartoon. (B) UMAP embedding on the latent space of the modified VEGA architecture colored by the experimental condition. (C) UMAP embedding on the latent space of the modified VEGA architecture colored by the activity of the INTERFERON SIGNALING pathway activity.

Supplementary Note 8:
We propose to adapt the VEGA architecture to the modelling of count data (SFig.8A), such as proposed by the scVI model[3]. For this purpose, an encoder representing the

latent library size scaling factor ℓ is added to the model, represented by a log-normal distribution approximated through variational inference similarly to the GMV encoder. The decoder is adapted to model a negative binomial (NB) distribution with parameter μ (represented by a VEGA sparse linear layer enforcing prior knowledge, with the addition of a softmax activation function at the output to encode the mean proportion of transcripts over all genes) and a gene-specific inverse dispersion θ represented as a set of learnable parameters. The loss function becomes the NB negative log-likelihood with two KL-divergence penalties for the GMV encoder parameters and the library size encoder parameters respectively (as described in[3]). This way, VEGA can be adapted to model count data while maintaining interpretability of the latent space in terms of GMV activity. We indeed show that such a model can be trained on the original count data of the Kang PBMC dataset using the Reactome database to initialize the GMVs, and interpretability is maintained in the latent space, as shown by the separation of control/stimulated cells by the interferon signaling activity (SFig.8B,C).

SFig.9: Training accuracy of logistic regression on VEGA's latent space for classifying control vs. stimulated cells in the Kang PBMC dataset. (A) Comparison of the training accuracy of a logistic regression model on 4 different loadings of VEGA and the Interpretable Autoencoder model proposed by Rybakov et al. (B) Runtime per epoch for VEGA and the Interpretable Autoencoder model.

Supplementary Note 9:
As a means to compare the information contained in VEGA's latent space in a similar way to Rybakov et al.[7], we trained a linear classifier (logistic regression) on a single GMV activity to classify stimulated cells vs. control cells in the Kang PBMC dataset (logistic regression trained respectively on CYTOKINE_SIGNALING_IN_IMMUNE_SYSTEM, ANTIVIRAL_MECHANISM_BY_IFN_STIMULATED_GENES, INTERFERON_ALPHA_BETA_SIGNALING, and INTERFERON_SIGNALING). We compared the training accuracy obtained on VEGA's GMV activities to those obtained on the loadings of the model proposed by Rybakov et al., trained with the same hyperparameters as VEGA. Specifically, we initialized models with the following parameters: VEGA: *add_nodes=1, dropout=0.5, z_dropout=0.5, positive_decoder=True*, Interpretable Autoencoder: *n_dense=1, n_sparse=0, dropout_rate=0.5, mid_layers_size=800*. Models were trained with the following hyperparameters: VEGA: *lr=1e-4, batch_size=128, n_epochs=500, train_patience=10.* For the Interpretable Autoencoder, we used the number of epochs at which VEGA stopped training (n=288) since the model doesn't implement early stopping: *lr=1e-4, batch_size=128, num_epochs=288, l2_reg_lambda0= 0.1, lambda1 = 0.93, lambda3 = 0.57.*

We show that VEGA performs comparatively to the Interpretable Autoencoder proposed by Rybakov et al. on this task (SFig.9A, average accuracy on VEGA: 0.98, average accuracy on Interpretable Autoencoder: 0.91) . We also compared the runtime of VEGA and the interpretable autoencoder model. We show that there is no major difference in the time to perform one epoch of the training process (SFig.9B).

SFig.10. Using soft constraints on VEGA's decoder to incorporate prior knowledge via L1-regularization can complete gene annotation knowledge in a data-driven fashion. Top 20 genes in the INTERFERON_ALPHA_BETA_SIGNALING pathway weight vector ranked by weight magnitude for L1-regularized versions of VEGA with regularization hyperparameter (A) $\lambda\eta$=1e-5, (B) $\lambda\eta$=1e-4, (C) $\lambda\eta$=1e-3, (D) $\lambda\eta$=1e-2, (E) $\lambda\eta$=1e-1.

Supplementary Note 10:
We sought to explore whether the masking procedure used in VEGA to incorporate prior knowledge from gene annotation databases could be relaxed and done via soft constraints such as regularization. This approach has been proposed by research parallel to ours by Rybakov et al.[7] and we propose to show here that it is complementary and beneficial to VEGA's modelling of GMVs. Instead of masking weights based on the matrix **M,** L1-regularization is used to penalize and induce sparsity for genes-pathway regulatory links for which no evidence is available in the gene annotation database (0 entries in **M**). Since the L1 regularization term is non-smooth, we use proximal gradient descent[8] to update the weights after the standard stochastic gradient descent learning step is taken for the convex penalty terms.

Since the L1-regularization term role is to enforce prior knowledge and potentially augment gene annotation databases, in order to choose the regularization hyperparameter λ and the learning rate η of the proximal gradient descent we decided to focus on recovering missing information from the gene annotation database rather than simply looking at the validation loss (as it would be done to determine hyperparameters in standard machine learning cross-validation procedures). We artificially removed 3 genes (*ISG15, ISG20, IFIT2*) from the "REACTOME_INTERFERON_ALPHA_BETA_SIGNALING" pathway of the Reactome pathway database. We then trained models on the Kang PBMC dataset using this truncated pathway reference for different values of the norm $\lambda\eta$ (which is used in the proximal gradient descent to update weights) : {1e-5, 1e-4, 1e-3, 1e-2, 1e-1}. The results are presented in SFig.10. We investigated the top 20 genes ranked by weights for the loading corresponding to the REACTOME_INTERFERON_ALPHA_BETA_SIGNALING to see how efficiently

pathway identity is enforced on the latent variables and how well it recovers the 3 genes that were artificially removed from the reference.
When $\lambda\eta$ is too small (1e-5, 1e-4, SFig.10A,B), prior knowledge about the pathway identity is not enforced strongly enough, as all the top genes are not part of the interferon alpha-beta signaling pathway. When $\lambda\eta$ is between 1e-3 and 1e-2 (SFig.10C,D), the model is able to recover the 3 missing genes artificially removed from the reference (denoted by stars *), as well as correctly enforcing pathway identity on the GMV. When $\lambda\eta$ is too large (1e-1, SFig.10D), pathway identity becomes strongly enforced and it is harder for the model to recover missing genes. The limit case where $\lambda\eta \rightarrow +\infty$ is equivalent to VEGA masking procedure, where the constraint on gene-pathway regulatory links absent from the database becomes too strong for the model to put any non-zero value on these weights.

SFig.11: Tradeoff between model performance and the biological signal encoded by GMVs when choosing the number of extra fully connected nodes. (A) Training and validation loss of the model as a function of the number of extra FC nodes in VEGA's latent space. Data are presented as mean values +/- standard deviation on 5 random model initialization and training. (B) Average silhouette width (ASW) computed on VEGA's latent space restricted to the GMV activities as a function of the number of extra FC nodes in VEGA's latent space. Data are presented as mean values +/- standard deviation on 5 random model initialization and training. The ASW was computed on different labels: experimental condition labels, cell type labels in the stimulated condition and cell type labels in the control condition.

Supplementary Note 11:
As VEGA allows the user to pick an arbitrary number of extra FC (fully connected) nodes to represent unannotated GMVs that explain remaining variance of the dataset, it is important to rationalize how many extra nodes are to be included in the latent space. When increasing the number of extra FC nodes, the model performance in terms of loss improves (SFig.11A). However, including too many extra FC nodes might encourage the model to only rely on those nodes for the encoding while disregarding the GMVs nodes. To verify this hypothesis, we computed the average silhouette score (ASW) on the latent space of the Kang PBCM dataset for the separation of the two conditions of the datasets, excluding the latent features corresponding to the extra FC nodes (SFig.11B). We report that the ASW decreases as more extra FC nodes are included in the modelling, suggesting that the information encoded by GMVs gets diluted as more extra FC nodes are added. The tradeoff between performance and informative GMVs encoding should guide the user in its choice of the number of extra FC nodes. We recommend avoiding including more than 16 extra FC in the model.

SFig.12: Including dropout in VEGA's latent space allows to model highly correlated GMVs by encouraging redundancy of latent variables. (A) Scatter plot of the interferon alpha beta signaling and interferon signaling activities in the Kang PBMCs dataset, inferred by VEGA trained with a latent dropout rate of 0 (blue) or 0.5 (orange). Plotted lines represent fitted regression lines. (B,C) Heatmaps of weights for the overlapping genes in the INTERFERON ALPHA BETA SIGNALING and INTERFERON SIGNALING pathways in VEGA's decoder, when trained with a a latent dropout rate of 0 (B) or 0.5 (C).

Supplementary Note 12:

As the diagonal covariance prior used in VEGA's latent space modelling discourages GMVs activities from being correlated, it is challenging to model the activity of pathways describing the same underlying biological processes (as certain gene annotation databases contain redundant information). To mitigate this, we propose to include dropout in the latent space of VEGA. This has been previously shown to allow the model to encode redundant information [9]. We demonstrate the effect of dropout in the latent variable mean / log-variance layers on the Kang PBMC dataset. It is expected that GMVs such as "Interferon alpha/beta signaling" and "Interferon signaling" should be correlated, since they describe the same underlying biological process. We demonstrate that the correlation between the activity of these two latent variables greatly improves when using a dropout layer in the latent space (dropout p=0.5, R^2=0.98), compared to no dropout at all (dropout p=0, R^2=0.06) (SFig.12A). When investigating the loadings for these two pathways in the linear decoder, we report that the weight vectors for the genes found in both pathways are more correlated when dropout is used (dropout p=0.5, R^2=1.00, SFig.12C) compared to no dropout (dropout p=0, R^2=0.72, SFig.12B), suggesting that the model can indeed encode redundant information when GMVs are expected to be correlated.

References:

1. Kang, H. M. *et al.* Multiplexed droplet single-cell RNA-sequencing using natural genetic variation. *Nat. Biotechnol.* **36**, 89–94 (2018).
2. Shekhar, K. *et al.* Comprehensive Classification of Retinal Bipolar Neurons by Single-Cell Transcriptomics. *Cell* **166**, 1308-1323.e30 (2016).
3. Lopez, R., Regier, J., Cole, M. B., Jordan, M. I. & Yosef, N. Deep generative modeling for single-cell transcriptomics. *Nat. Methods* **15**, 1053–1058 (2018).
4. Svensson, V., Gayoso, A., Yosef, N. & Pachter, L. Interpretable factor models of single-cell

RNA-seq via variational autoencoders. *Bioinformatics* **36**, 3418–3421 (2020).

5. McFarland, J. M. *et al.* Multiplexed single-cell transcriptional response profiling to define cancer vulnerabilities and therapeutic mechanism of action. *Nat. Commun.* **11**, 4296 (2020).
6. Marczyk, M. *et al.* Multi-Omics Investigation of Innate Navitoclax Resistance in Triple-Negative Breast Cancer Cells. *Cancers* **12**, 2551 (2020).
7. Rybakov, S., Lotfollahi, M., Theis, F. J. & Wolf, F. A. *Learning interpretable latent autoencoder representations with annotations of feature sets*. http://biorxiv.org/lookup/doi/10.1101/2020.12.02.401182 (2020) doi:10.1101/2020.12.02.401182.
8. Proximal Algorithms | Foundations and Trends in Optimization. https://dl.acm.org/doi/abs/10.1561/2400000003.
9. Yeung, S., Kannan, A., Dauphin, Y. & Fei-Fei, L. EPITOMIC VARIATIONAL AUTOENCODER. 16 (2017).
10. Buettner, F., Pratanwanich, N., McCarthy, D. J., Marioni, J. C. & Stegle, O. f-scLVM: scalable and versatile factor analysis for single-cell RNA-seq. *Genome Biol.* **18**, 212 (2017).

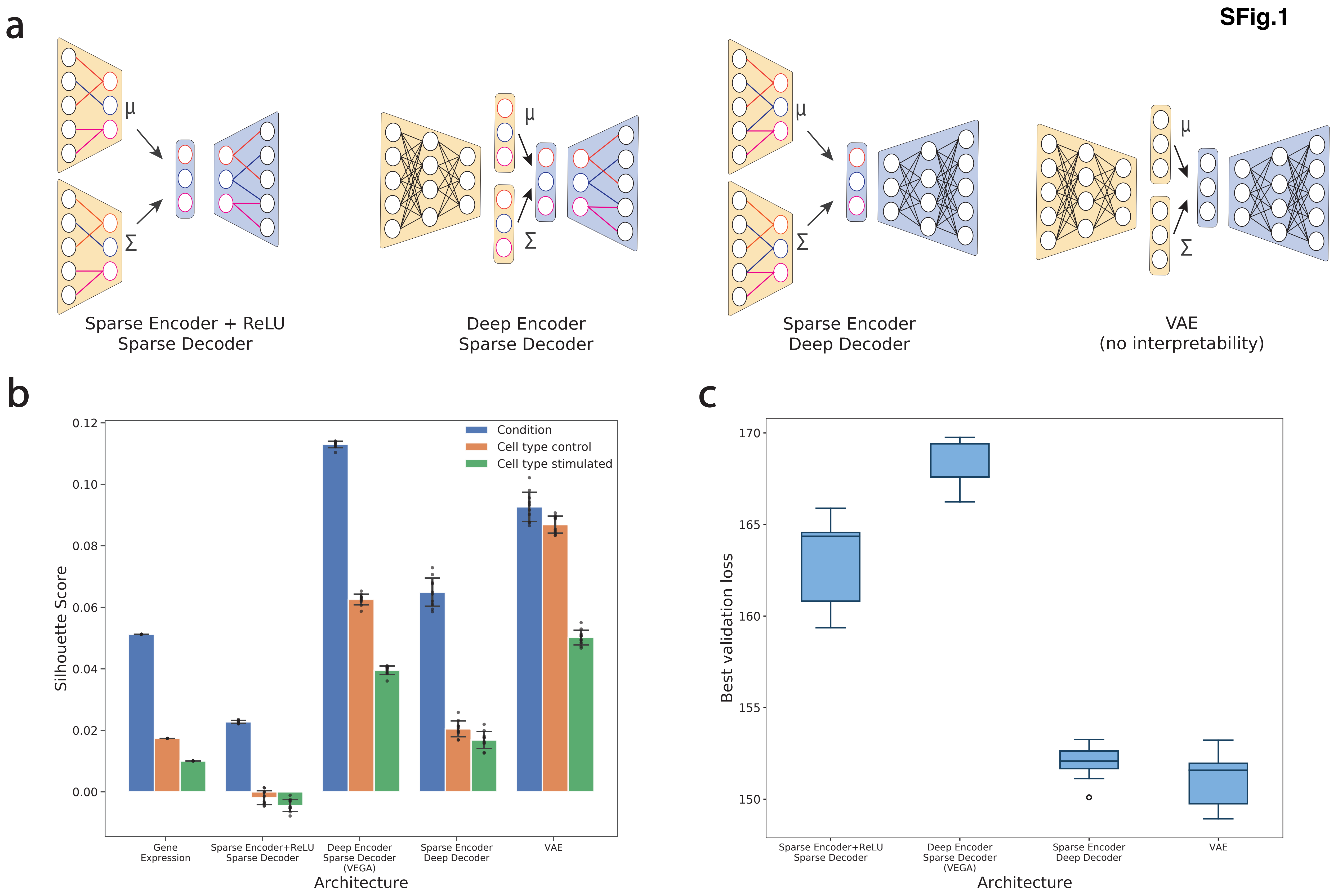
SFig.1
a
μ
Σ
Sparse Encoder + ReLU
Sparse Decoder
Deep Encoder
Sparse Decoder
Sparse Encoder
Deep Decoder
VAE
(no interpretability)
b
Condition
Cell type control
Cell type stimulated
Silhouette Score
0.12
0.10
0.08
0.06
0.04
0.02
0.00
Gene Expression
Sparse Encoder+ReLU Sparse Decoder
Deep Encoder Sparse Decoder (VEGA)
Sparse Encoder Deep Decoder
VAE
Architecture
c
Best validation loss
170
165
160
155
150
Sparse Encoder+ReLU Sparse Decoder
Deep Encoder Sparse Decoder (VEGA)
Sparse Encoder Deep Decoder
VAE
Architecture

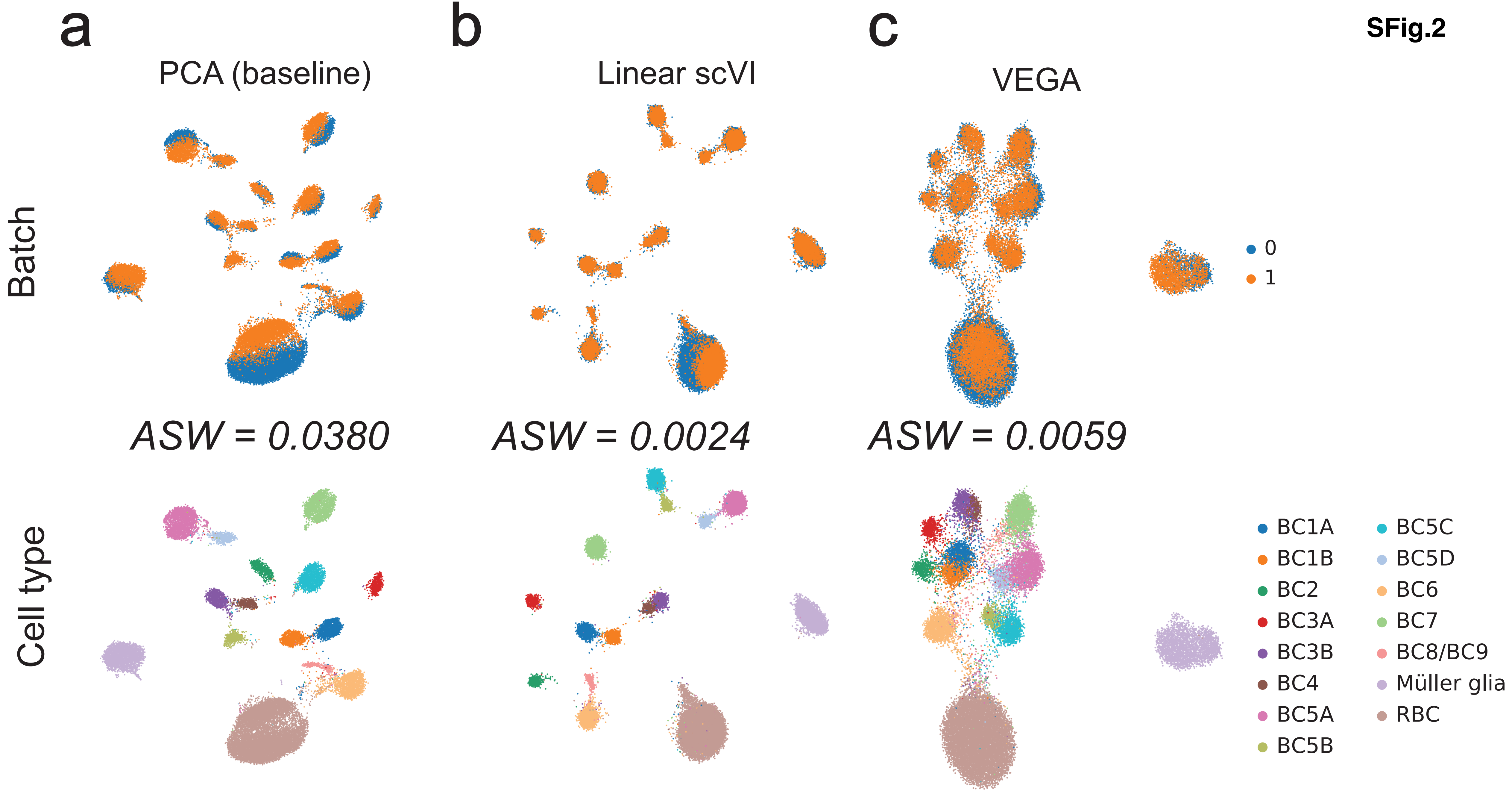

SFig.2
a
PCA (baseline)
b
Linear scVI
c
VEGA
Batch
0
1
ASW = 0.0380
ASW = 0.0024
ASW = 0.0059
Cell type
BC1A
BC1B
BC2
BC3A
BC3B
BC4
BC5A
BC5B
BC5C
BC5D
BC6
BC7
BC8/BC9
Müller glia
RBC

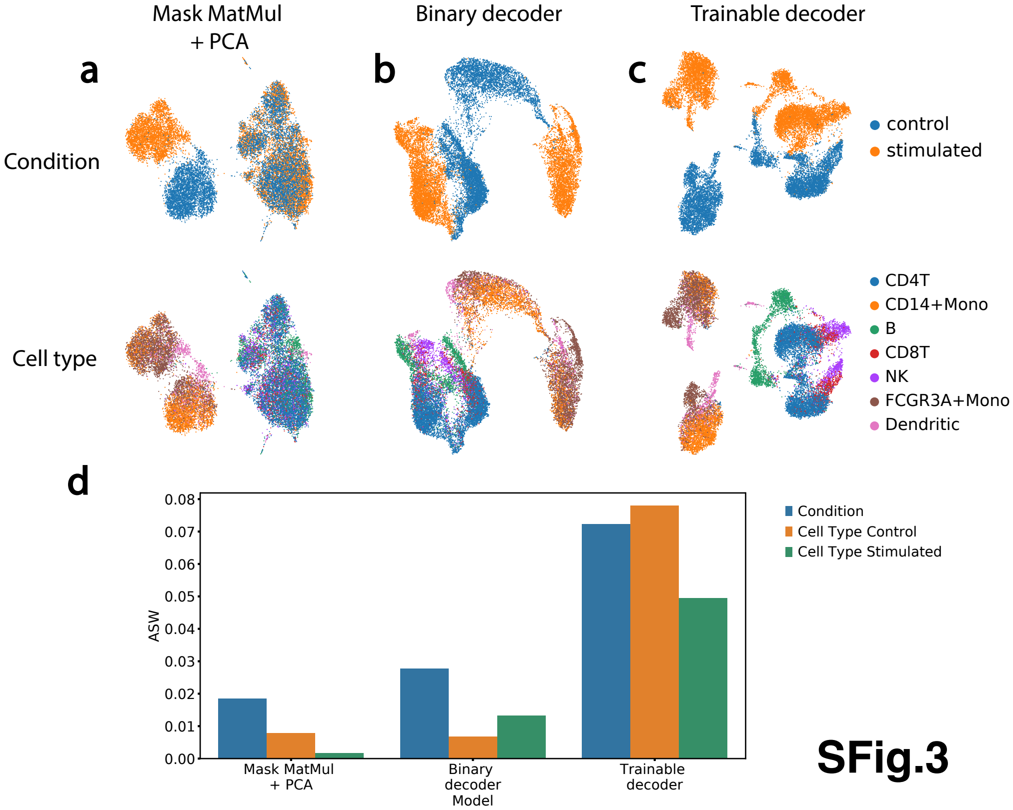

Mask MatMul
+ PCA
Binary decoder
Trainable decoder
a
b
c
Condition
control
stimulated
Cell type
CD4T
CD14+Mono
B
CD8T
NK
FCGR3A+Mono
Dendritic
d
ASW
0.08
0.07
0.06
0.05
0.04
0.03
0.02
0.01
0.00
Condition
Cell Type Control
Cell Type Stimulated
Mask MatMul
+ PCA
Binary
decoder
Trainable
decoder
Model

SFig.3

SFig.4

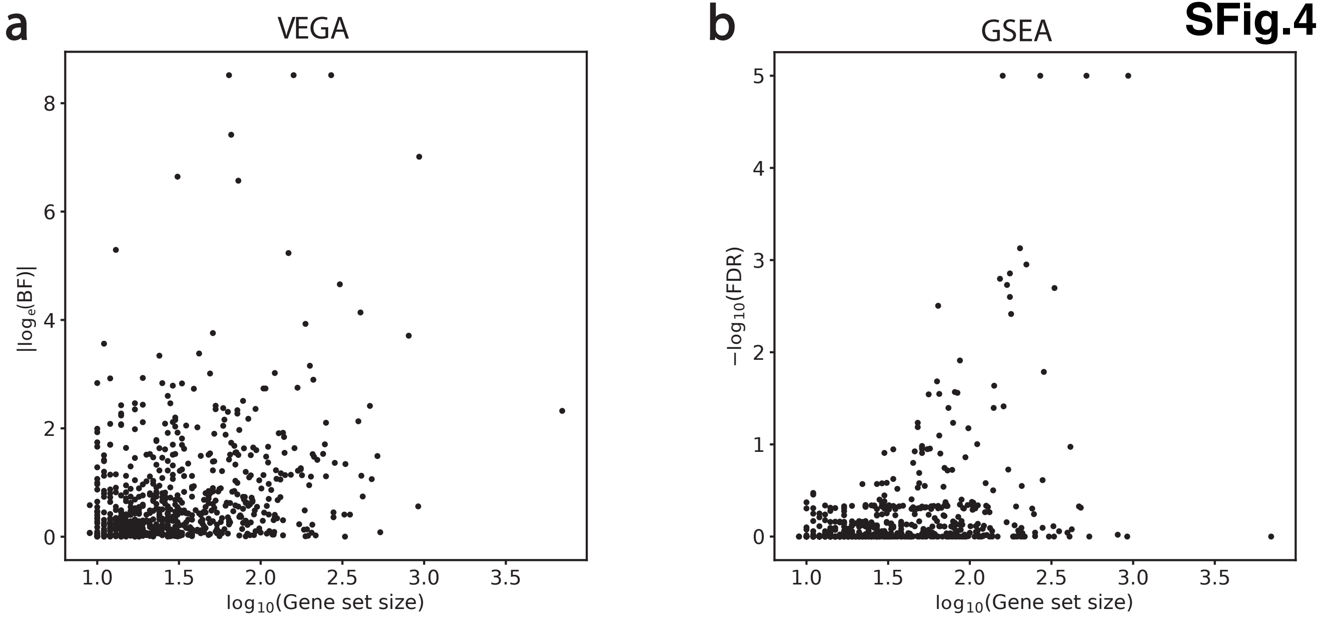

a
VEGA
|log_e(BF)|
log10(Gene set size)
b
GSEA
−log10(FDR)
log10(Gene set size)

SFig.5

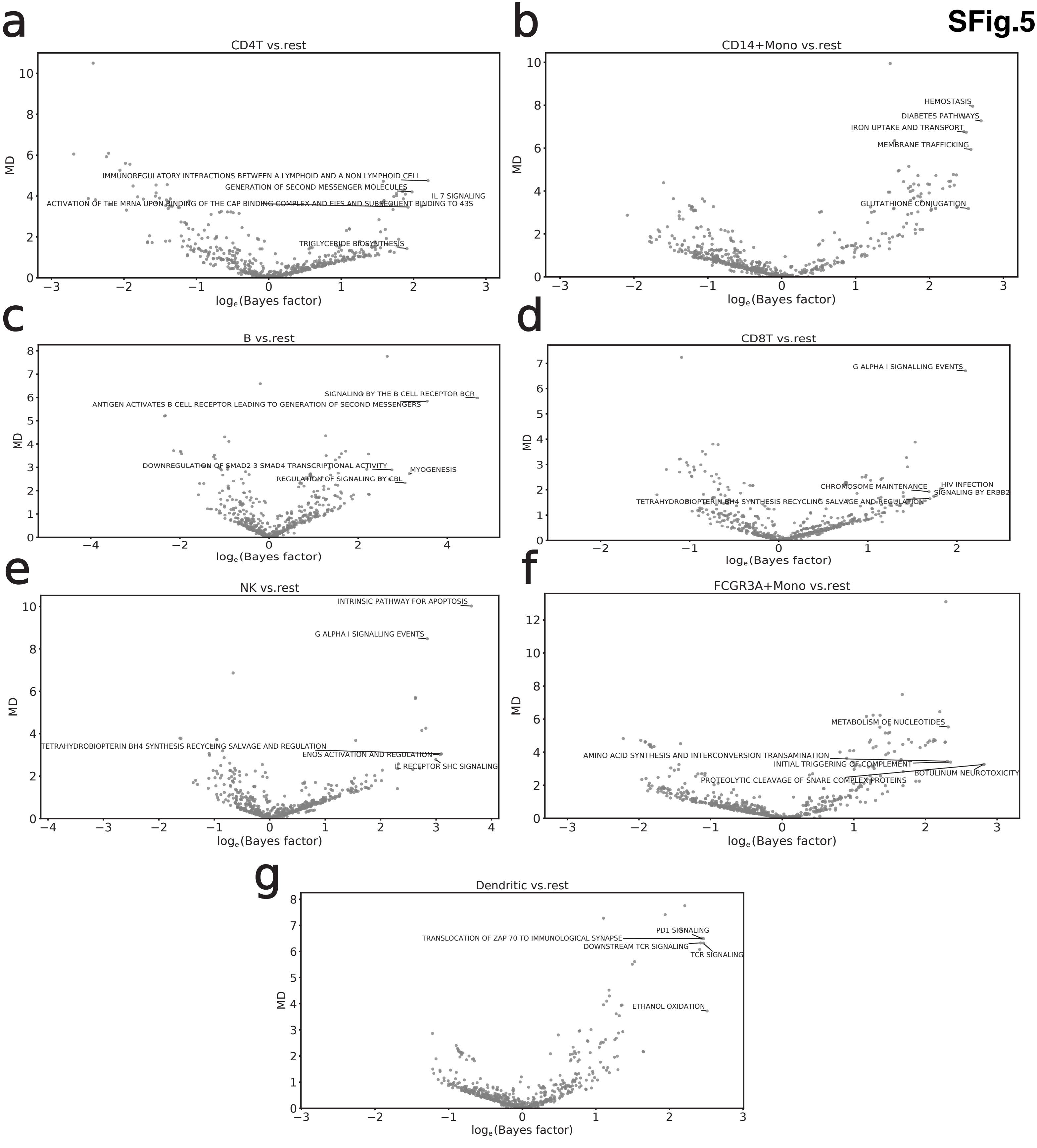
a
CD4T vs.rest
IMMUNOREGULATORY INTERACTIONS BETWEEN A LYMPHOID AND A NON LYMPHOID CELL
GENERATION OF SECOND MESSENGER MOLECULES
IL 7 SIGNALING
ACTIVATION OF THE MRNA UPON BINDING OF THE CAP BINDING COMPLEX AND EIFS AND SUBSEQUENT BINDING TO 43S
TRIGLYCERIDE BIOSYNTHESIS
MD
log_e(Bayes factor)
b
CD14+Mono vs.rest
HEMOSTASIS
DIABETES PATHWAYS
IRON UPTAKE AND TRANSPORT
MEMBRANE TRAFFICKING
GLUTATHIONE CONJUGATION
MD
log_e(Bayes factor)
c
B vs.rest
SIGNALING BY THE B CELL RECEPTOR BCR
ANTIGEN ACTIVATES B CELL RECEPTOR LEADING TO GENERATION OF SECOND MESSENGERS
DOWNREGULATION OF SMAD2 3 SMAD4 TRANSCRIPTIONAL ACTIVITY
MYOGENESIS
REGULATION OF SIGNALING BY CBL
MD
log_e(Bayes factor)
d
CD8T vs.rest
G ALPHA I SIGNALLING EVENTS
CHROMOSOME MAINTENANCE
HIV INFECTION
SIGNALING BY ERBB2
TETRAHYDROBIOPTERIN BH4 SYNTHESIS RECYCLING SALVAGE AND REGULATION
MD
log_e(Bayes factor)
e
NK vs.rest
INTRINSIC PATHWAY FOR APOPTOSIS
G ALPHA I SIGNALLING EVENTS
TETRAHYDROBIOPTERIN BH4 SYNTHESIS RECYCLING SALVAGE AND REGULATION
ENOS ACTIVATION AND REGULATION
IL RECEPTOR SHC SIGNALING
MD
log_e(Bayes factor)
f
FCGR3A+Mono vs.rest
METABOLISM OF NUCLEOTIDES
AMINO ACID SYNTHESIS AND INTERCONVERSION TRANSAMINATION
INITIAL TRIGGERING OF COMPLEMENT
BOTULINUM NEUROTOXICITY
PROTEOLYTIC CLEAVAGE OF SNARE COMPLEX PROTEINS
MD
log_e(Bayes factor)
g
Dendritic vs.rest
PD1 SIGNALING
TRANSLOCATION OF ZAP 70 TO IMMUNOLOGICAL SYNAPSE
DOWNSTREAM TCR SIGNALING
TCR SIGNALING
ETHANOL OXIDATION
MD
log_e(Bayes factor)

SFig.6

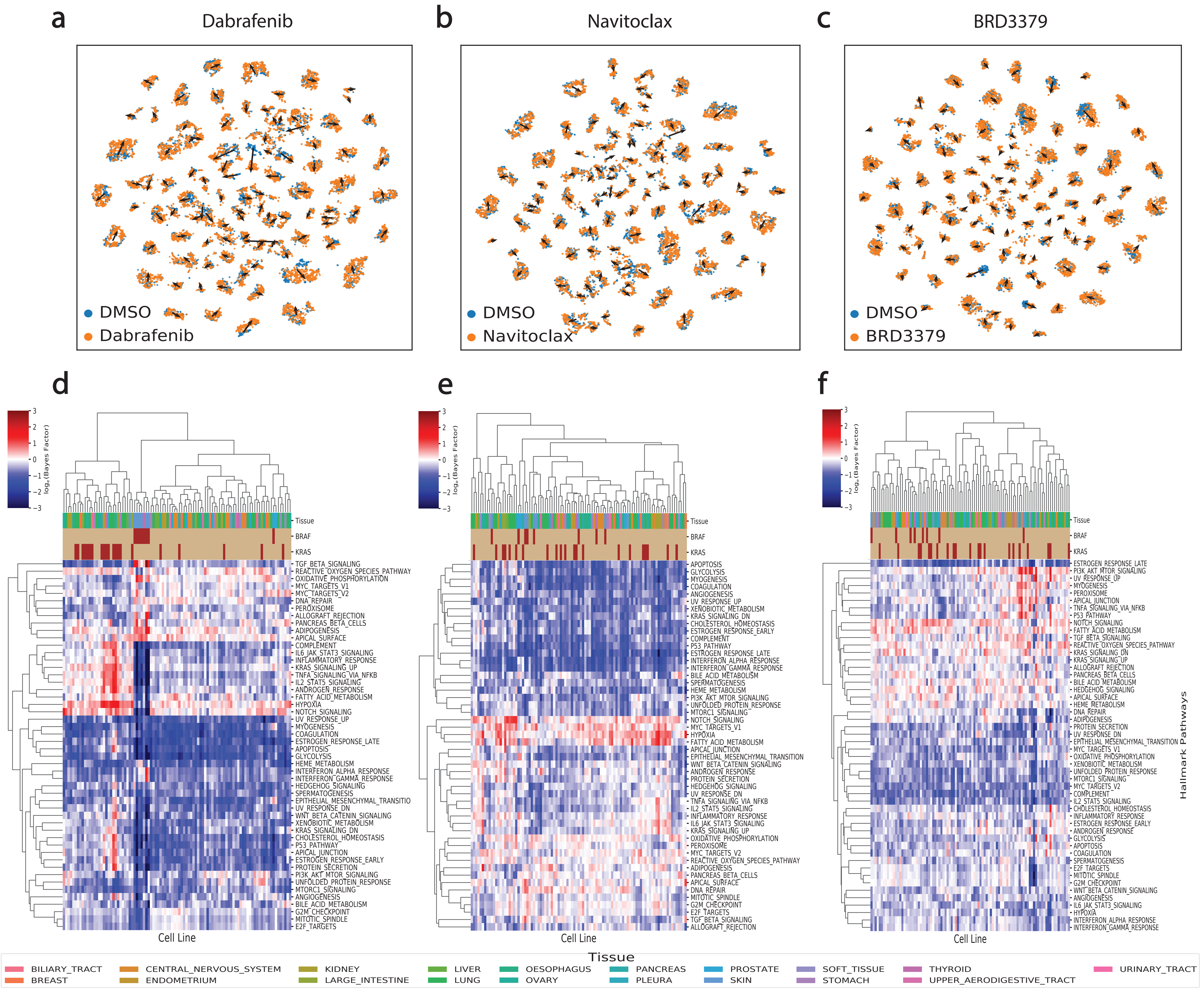
a
Dabrafenib
DMSO
Dabrafenib
b
Navitoclax
DMSO
Navitoclax
c
BRD3379
DMSO
BRD3379
d
e
f
loge(Bayes Factor)
Tissue
BRAF
KRAS
TGF_BETA_SIGNALING
REACTIVE_OXYGEN_SPECIES_PATHWAY
OXIDATIVE_PHOSPHORYLATION
MYC_TARGETS_V1
MYC_TARGETS_V2
DNA_REPAIR
PEROXISOME
ALLOGRAFT_REJECTION
PANCREAS_BETA_CELLS
ADIPOGENESIS
APICAL_SURFACE
IL6_JAK_STAT3_SIGNALING
INFLAMMATORY_RESPONSE
KRAS_SIGNALING_UP
TNFA_SIGNALING_VIA_NFKB
IL2_STAT5_SIGNALING
ANDROGEN_RESPONSE
FATTY_ACID_METABOLISM
HYPOXIA
NOTCH_SIGNALING
UV_RESPONSE_UP
MYOGENESIS
COAGULATION
ESTROGEN_RESPONSE_LATE
APOPTOSIS
GLYCOLYSIS
HEME_METABOLISM
INTERFERON_ALPHA_RESPONSE
INTERFERON_GAMMA_RESPONSE
HEDGEHOG_SIGNALING
SPERMATOGENESIS
EPITHELIAL_MESENCHYMAL_TRANSITIO
UV_RESPONSE_DN
WNT_BETA_CATENIN_SIGNALING
XENOBIOTIC_METABOLISM
KRAS_SIGNALING_DN
CHOLESTEROL_HOMEOSTASIS
P53_PATHWAY
APICAL_JUNCTION
ESTROGEN_RESPONSE_EARLY
PROTEIN_SECRETION
PI3K_AKT_MTOR_SIGNALING
UNFOLDED_PROTEIN_RESPONSE
MTORC1_SIGNALING
ANGIOGENESIS
BILE_ACID_METABOLISM
G2M_CHECKPOINT
MITOTIC_SPINDLE
E2F_TARGETS
Cell Line
Hallmark Pathways
Tissue
BILIARY_TRACT
BREAST
CENTRAL_NERVOUS_SYSTEM
ENDOMETRIUM
KIDNEY
LARGE_INTESTINE
LIVER
LUNG
OESOPHAGUS
OVARY
PANCREAS
PLEURA
PROSTATE
SKIN
SOFT_TISSUE
STOMACH
THYROID
UPPER_AERODIGESTIVE_TRACT
URINARY_TRACT

SFig.7

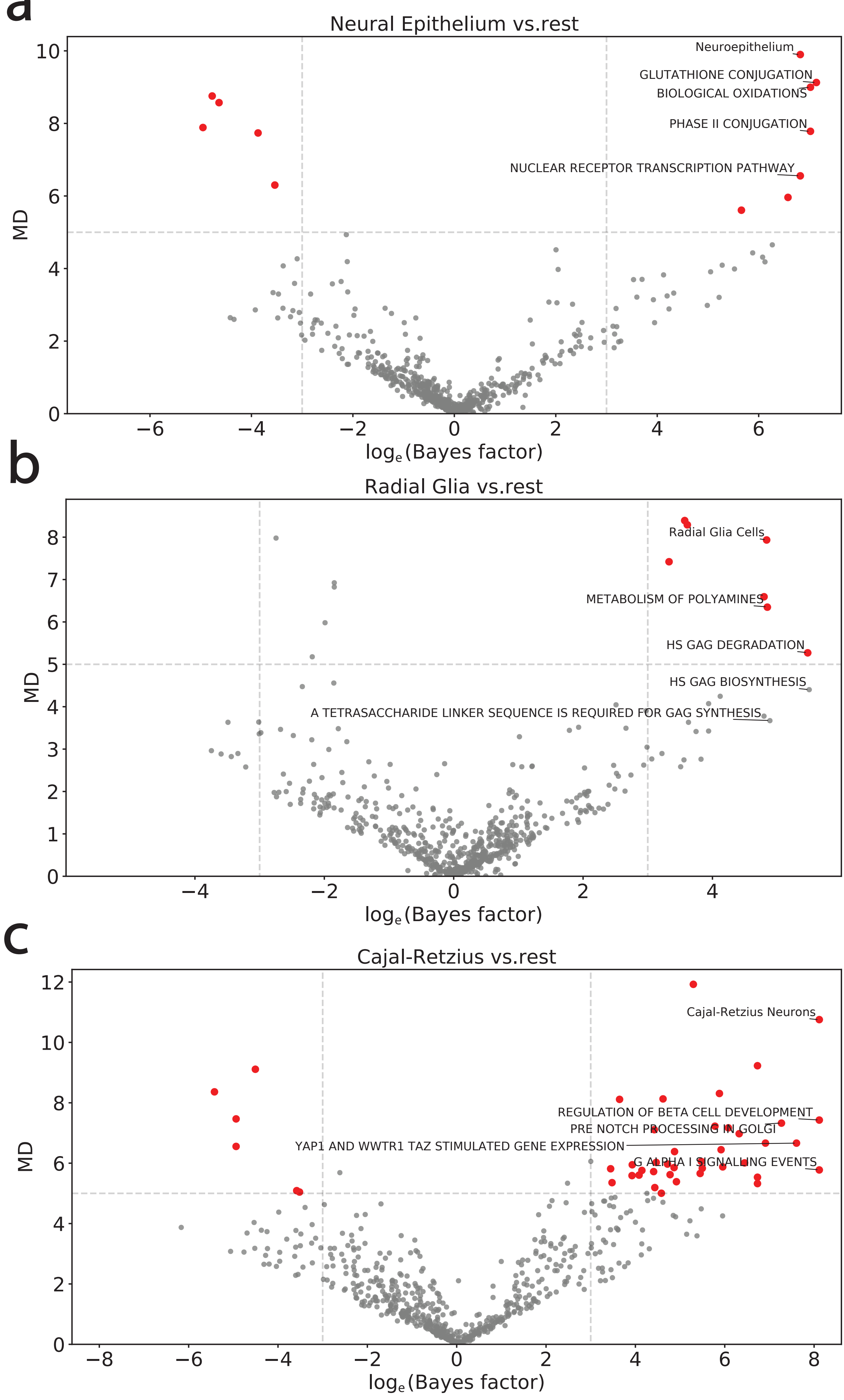
a
Neural Epithelium vs.rest
Neuroepithelium
GLUTATHIONE CONJUGATION
BIOLOGICAL OXIDATIONS
PHASE II CONJUGATION
NUCLEAR RECEPTOR TRANSCRIPTION PATHWAY
MD
log_e(Bayes factor)
b
Radial Glia vs.rest
Radial Glia Cells
METABOLISM OF POLYAMINES
HS GAG DEGRADATION
HS GAG BIOSYNTHESIS
A TETRASACCHARIDE LINKER SEQUENCE IS REQUIRED FOR GAG SYNTHESIS
MD
log_e(Bayes factor)
c
Cajal-Retzius vs.rest
Cajal-Retzius Neurons
REGULATION OF BETA CELL DEVELOPMENT
PRE NOTCH PROCESSING IN GOLGI
YAP1 AND WWTR1 TAZ STIMULATED GENE EXPRESSION
G ALPHA I SIGNALLING EVENTS
MD
log_e(Bayes factor)

SFig.8

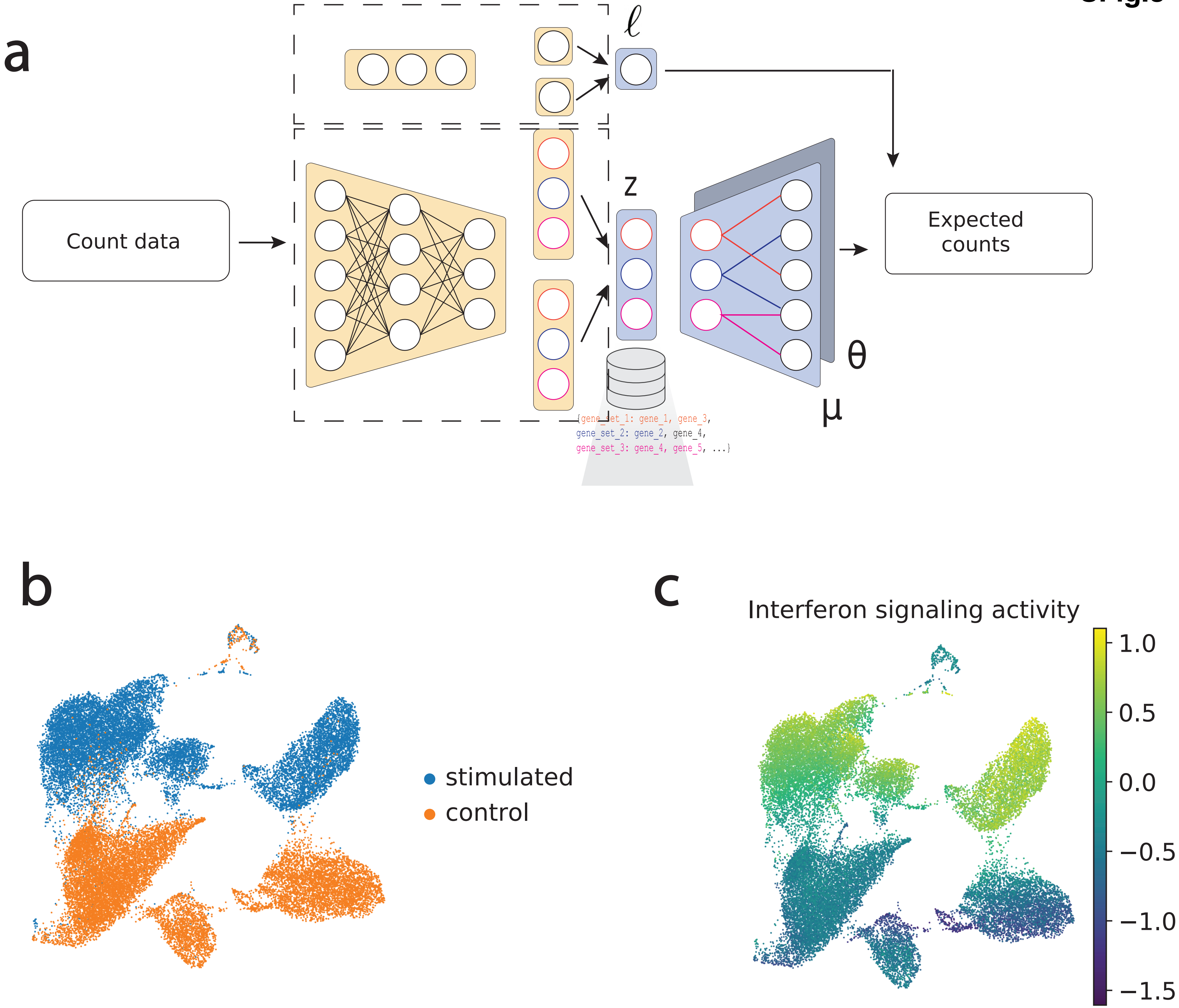
a
ℓ
z
Count data
Expected
counts
θ
μ
{gene_set_1: gene_1, gene_3,
gene_set_2: gene_2, gene_4,
gene_set_3: gene_4, gene_5, ...}
b
stimulated
control
c
Interferon signaling activity
1.0
0.5
0.0
−0.5
−1.0
−1.5

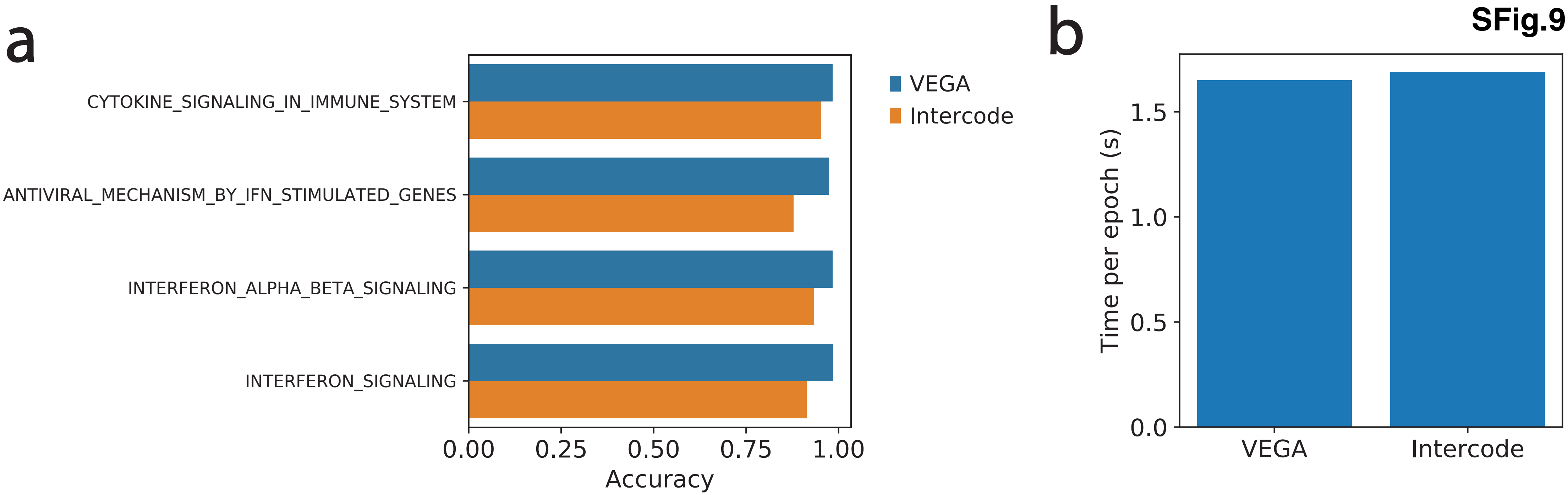
a
CYTOKINE_SIGNALING_IN_IMMUNE_SYSTEM
ANTIVIRAL_MECHANISM_BY_IFN_STIMULATED_GENES
INTERFERON_ALPHA_BETA_SIGNALING
INTERFERON_SIGNALING
0.00
0.25
0.50
0.75
1.00
Accuracy
VEGA
Intercode
b
Time per epoch (s)
0.0
0.5
1.0
1.5
VEGA
Intercode

SFig.10

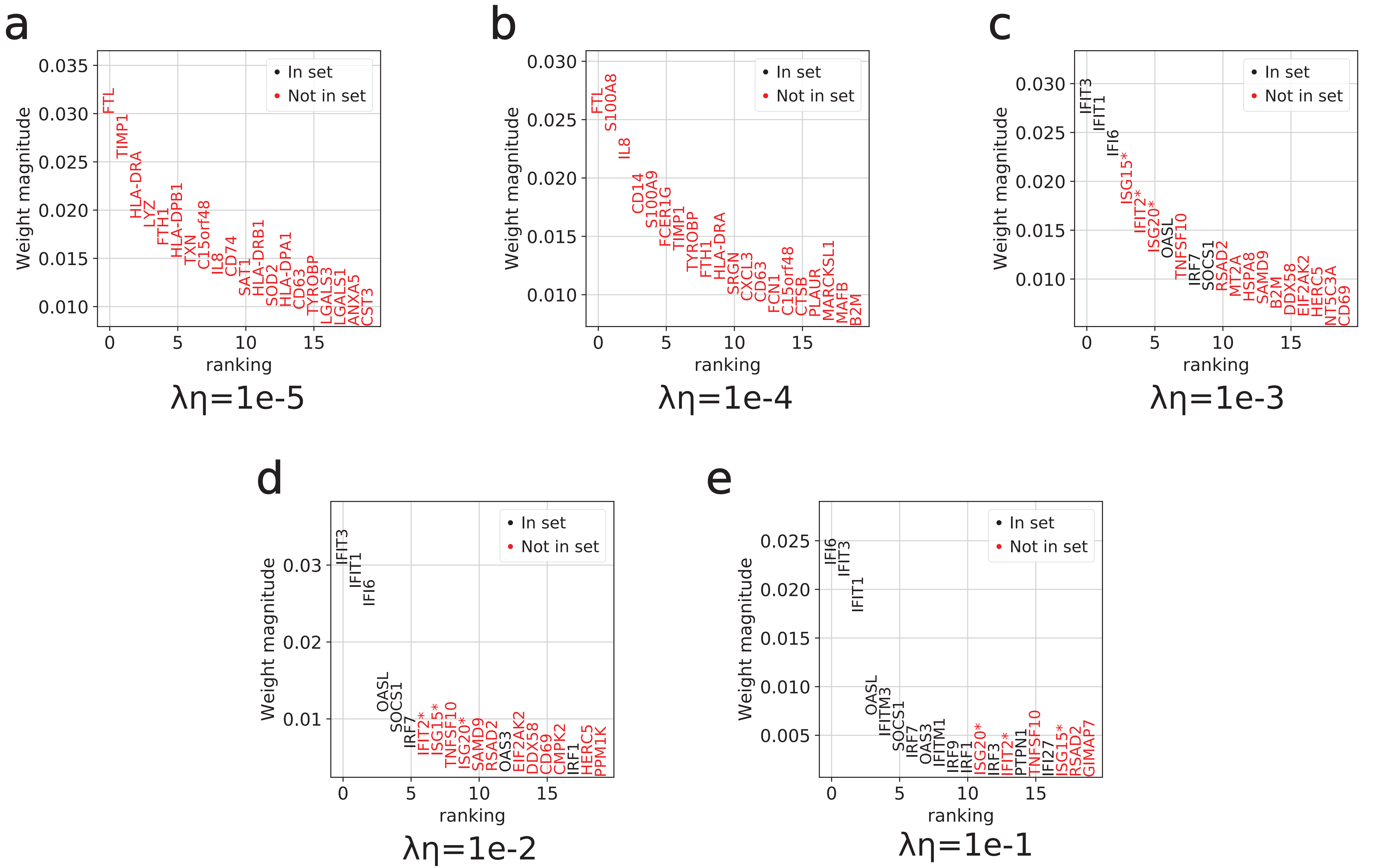

a
b
c
d
e
Weight magnitude
ranking
In set
Not in set
λη=1e-5
λη=1e-4
λη=1e-3
λη=1e-2
λη=1e-1
FTL
TIMP1
HLA-DRA
LYZ
FTH1
HLA-DPB1
TXN
C15orf48
IL8
CD74
SAT1
HLA-DRB1
SOD2
HLA-DPA1
CD63
TYROBP
LGALS3
LGALS1
ANXA5
CST3
S100A8
CD14
S100A9
FCER1G
SRGN
CXCL3
FCN1
CTSB
PLAUR
MARCKSL1
MAFB
B2M
IFIT3
IFIT1
IFI6
ISG15*
IFIT2*
ISG20*
OASL
TNFSF10
IRF7
SOCS1
RSAD2
MT2A
HSPA8
SAMD9
DDX58
EIF2AK2
HERC5
NT5C3A
CD69
OAS3
IRF1
CMPK2
PPM1K
IFITM3
IFITM1
IRF9
IRF3
PTPN1
IFI27
GIMAP7

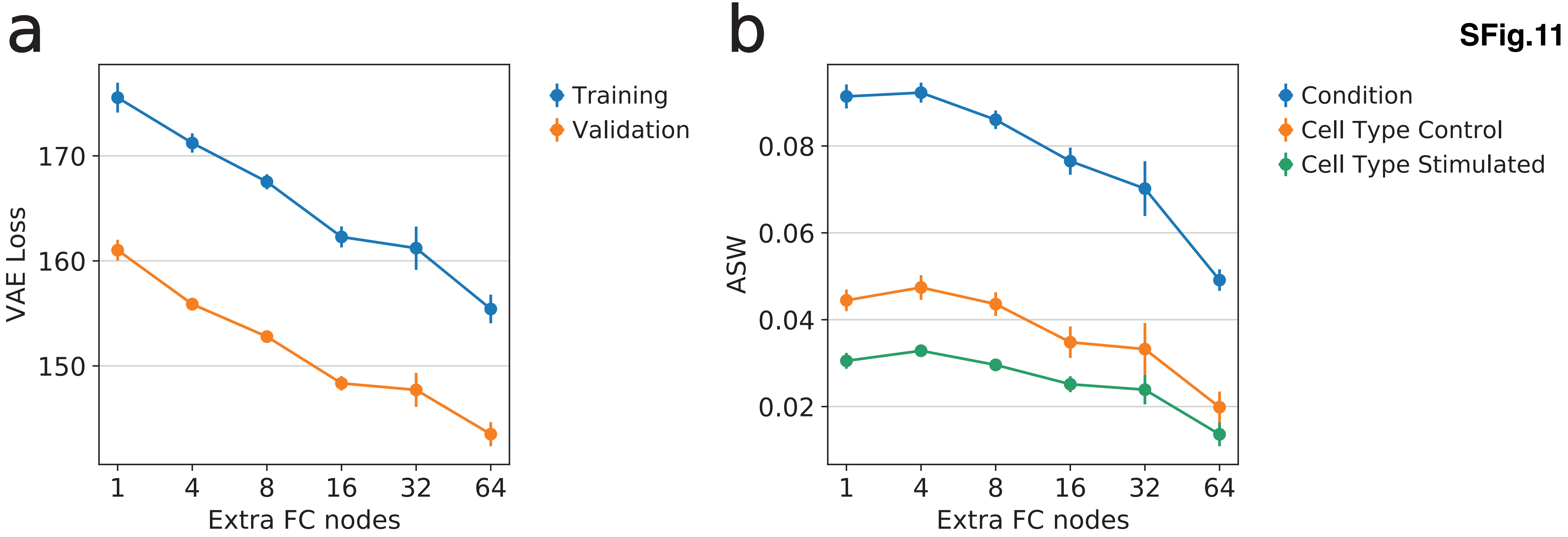

a
b
SFig.11
Training
Validation
VAE Loss
170
160
150
1
4
8
16
32
64
Extra FC nodes
Condition
Cell Type Control
Cell Type Stimulated
ASW
0.08
0.06
0.04
0.02
1
4
8
16
32
64
Extra FC nodes

SFig.12

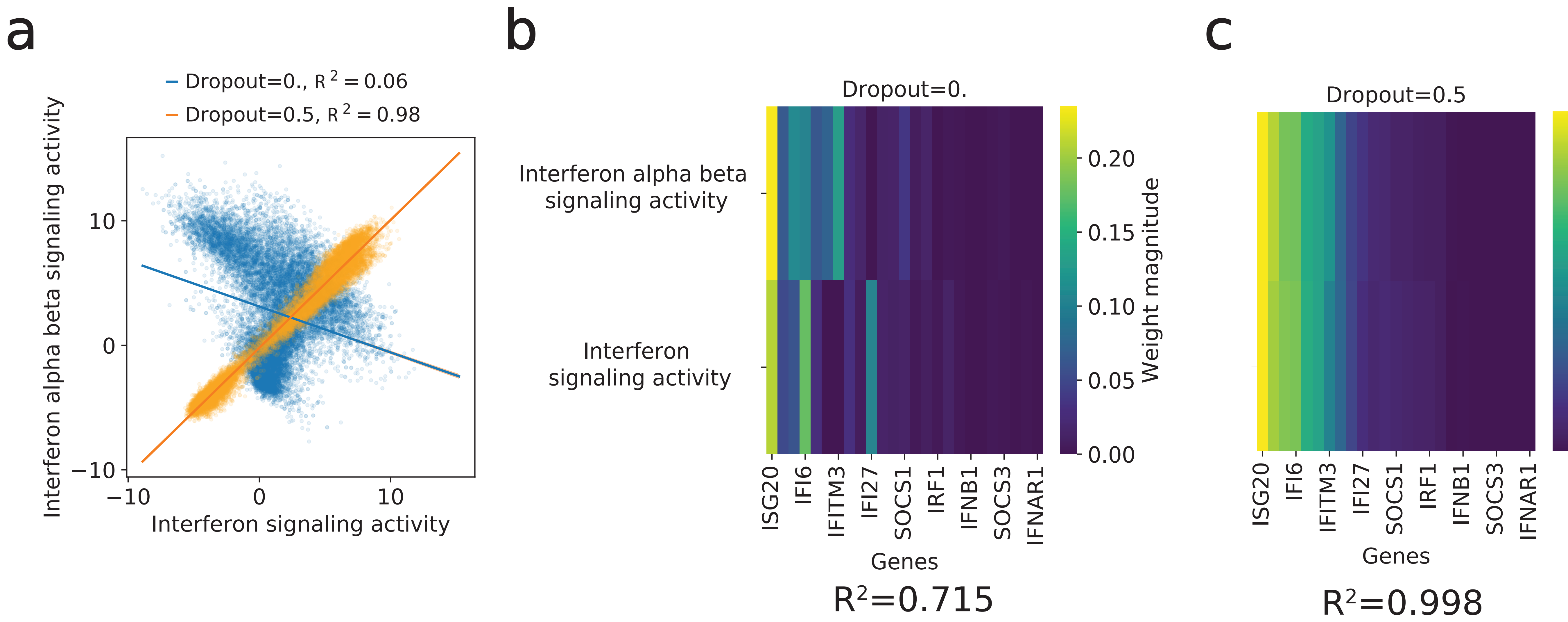

a
Dropout=0., $R^2 = 0.06$
Dropout=0.5, $R^2 = 0.98$
Interferon alpha beta signaling activity
Interferon signaling activity
10
0
−10
−10
0
10
b
Dropout=0.
Interferon alpha beta signaling activity
Interferon signaling activity
0.20
0.15
0.10
0.05
0.00
Weight magnitude
ISG20
IFI6
IFITM3
IFI27
SOCS1
IRF1
IFNB1
SOCS3
IFNAR1
Genes
R^2=0.715
c
Dropout=0.5
0.04
0.03
0.02
0.01
0.00
Weight magnitude
ISG20
IFI6
IFITM3
IFI27
SOCS1
IRF1
IFNB1
SOCS3
IFNAR1
Genes
R^2=0.998
