# Supplementary material for: VEGA is an interpretable generative model for inferring biological network activity in single-cell transcriptomics
Source: Nat Commun. 2021 Sep 28;12:5684. doi: 10.1038/s41467-021-26017-0 (PMC8478947; doi:10.1038/s41467-021-26017-0)
Supplement: Supplementary file 2 — Description of Additional Supplementary Files [file 41467_2021_26017_MOESM2_ESM.pdf]

File Name: Supplementary Data 1
Description: Results for differentially GMV activation analysis in the context of stimulated vs. control immune cells.

File Name: Supplementary Data 2
Description: Results for differentially GMV activation analysis in the context of early brain development.
